# Liver Stiffness Directs Intrahepatic Cholesterol Accumulation Through YAP/TAZ in Metabolic Dysfunction‐Associated Steatotic Liver Disease

**DOI:** 10.1002/advs.202500379

**Published:** 2026-02-26

**Authors:** Na Young Lee, Myeung Gi Choi, Ho Jae Ryu, Young Jin Min, Seon Min Kim, Yeonseok Chung, Bo Kyung Koo, Yun Pyo Kang, Won Kim, Ja Hyun Koo

**Affiliations:** ^1^ College of Pharmacy Seoul National University Seoul Republic of Korea; ^2^ Department of Internal Medicine Seoul National University College of Medicine Seoul Republic of Korea; ^3^ Division of Endocrinology and Metabolism Department of Internal Medicine Seoul Metropolitan Government Boramae Medical Center Seoul Republic of Korea; ^4^ Division of Gastroenterology and Hepatology Department of Internal Medicine Seoul Metropolitan Government Boramae Medical Center Seoul Republic of Korea; ^5^ Research Institute of Pharmaceutical Sciences and Natural Products Research Institute College of Pharmacy Seoul National University Seoul Republic of Korea

**Keywords:** liver stiffness, LXRα, MASLD, YAP/TAZ

## Abstract

Elevated liver stiffness is closely associated with morbidity and mortality in metabolic dysfunction‐associated steatotic liver disease (MASLD). However, the contribution of increased stiffness to impaired liver function is poorly understood. Here, we demonstrate that hepatic cholesterol levels are determined by the stiffness of the liver. In the human MASLD cohort and a mouse model, intrahepatic cholesterol levels strongly correlated with liver stiffness. We show that a stiff matrix promotes spontaneous accumulation of cholesterol in isolated hepatocytes. As the underlying mechanism, we found that Liver X receptor alpha (LXRα) is mechanosensitively repressed. Activation of Yes‐associated protein (YAP) and Transcriptional coactivator with PDZ‐binding motif (TAZ) by exposure to stiff substrate, serum stimulation, low‐density culture, or deletion of Large tumor suppressor kinase 1 and 2 (LATS1/2) robustly repressed LXRα activity. In the nucleus, YAP disrupted heterodimerization of LXRα with Retinoid X receptor alpha (RXRα) independently of their transcriptional activity. Consistently, hepatocyte‐specific ablation of Yap/Taz facilitated hepatic cholesterol efflux and delayed cholesterol‐induced fibrosis progression in mice. Transcriptomic analysis of MASLD patient livers confirmed a strong inverse correlation between LXRα target gene expression and liver stiffness as well as YAP/TAZ activity. These findings reveal the mechanosensitive regulation of hepatic cholesterol levels in MASLD, suggesting liver stiffness as a causal factor for hepatocyte dysfunction.

## Graphical abstract

Liver stiffness promotes intrahepatic cholesterol accumulation by repressing LXRα through YAP/TAZ activation. Stiff matrices impair cholesterol efflux in hepatocytes, while YAP/TAZ deletion restores LXRα activity and prevents cholesterol‐induced fibrosis. Analyses of MASLD patients reveal inverse correlations between liver stiffness and hepatic cholesterol levels, and between LXRα target expression and stiffness, defining a mechanotransductive cycle linking fibrosis with metabolic dysfunction.

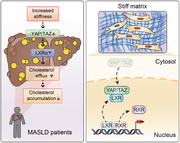

## 1 Introduction

Metabolic dysfunction‐associated steatotic liver disease (MASLD) has emerged as the most prevalent chronic disease, currently affecting one‐third of the global population [1, 2]. MASLD encompasses a wide range of spectra from steatosis and steatohepatitis to fibrosis and ultimately cirrhosis, where liver fibrosis marks the onset of significant hepatocyte dysfunction and serves as a primary predictor of mortality and liver‐related events [3]. Despite extensive research, effective therapeutic strategies remain limited, as resmetirom and semaglutide are the only approved therapies for MASLD and confer only modest anti‐fibrotic benefits [4].

A critical but underexplored aspect of MASLD pathophysiology involves the mechanical properties of the liver. Liver stiffness has been widely accepted as a standard diagnostic marker for MASLD severity, particularly with the advent of vibration‐controlled transient elastography, a non‐invasive and cost‐effective alternative to liver biopsy [5, 6]. Healthy liver tissue exhibits remarkable softness (1–3 kPa), but progressive fibrosis can increase stiffness by more than ten‐fold (>20 kPa) [7, 8]. While liver stiffness measurement via transient elastography has become the gold standard for non‐invasive fibrosis assessment, the biological consequences of this mechanical transformation on hepatocyte function have received limited attention.

Recent advances in mechanotransduction signaling influence a wide range of cellular functions, including metabolism, differentiation, and proliferation [9]. The transcriptional coactivators Yes‐associated protein (YAP) and Transcriptional coactivator with PDZ‐binding motif (TAZ) are central mechanosensors that mediate cellular responses to mechanical cues such as matrix stiffness, cell density, stretching, and pressure loading [10]. In response to stiff matrix, YAP/TAZ translocate to the nucleus and alter gene transcription through association with their transcriptional partners, representatively TEAD [10]. In the adult liver, YAP/TAZ activity is mainly restricted to cholangiocytes, but enhanced activity of YAP/TAZ in hepatocytes is commonly observed in fibrotic livers of patients and experimental mouse models [11, 12]. Therefore, it is plausible that hepatocytes are also able to respond to matrix stiffness, particularly during liver fibrosis progression. Nonetheless, whether hepatocytes can directly sense matrix stiffness and the link between liver stiffness and hepatocyte dysfunction remains largely unexplored.

In the liver, cellular cholesterol is a pivotal factor for MASLD progression [13, 14, 15]. One of the most prominent hepatic cholesterol regulators is Liver X receptor alpha (LXRα), a liver‐enriched nuclear receptor [16]. The absence or impaired activity of LXRα leads to cholesterol accumulation and liver damage in MASLD [17, 18]. However, effective approaches for the control of hepatic cholesterol have not been achieved, and the endogenous cues responsible for cholesterol accumulation in MASLD remain to be elucidated.

The intersection of mechanobiology and cholesterol metabolism has been largely unexplored in the context of liver disease. However, a recent study has suggested that cholesterol serves as an upstream input activating TAZ [19]. This finding has led us to expect that activated YAP/TAZ by the stiff matrix in MASLD may also play a role in actively controlling intracellular cholesterol levels, which has yet to be investigated. Here, we hypothesize that liver stiffness directly influences hepatocyte cholesterol metabolism through YAP/TAZ‐mediated regulation of LXRα activity.

## 2 Results

### 2.1 Hepatic Cholesterol Content Correlates with Tissue Stiffness in MASLD

**FIGURE 1:**
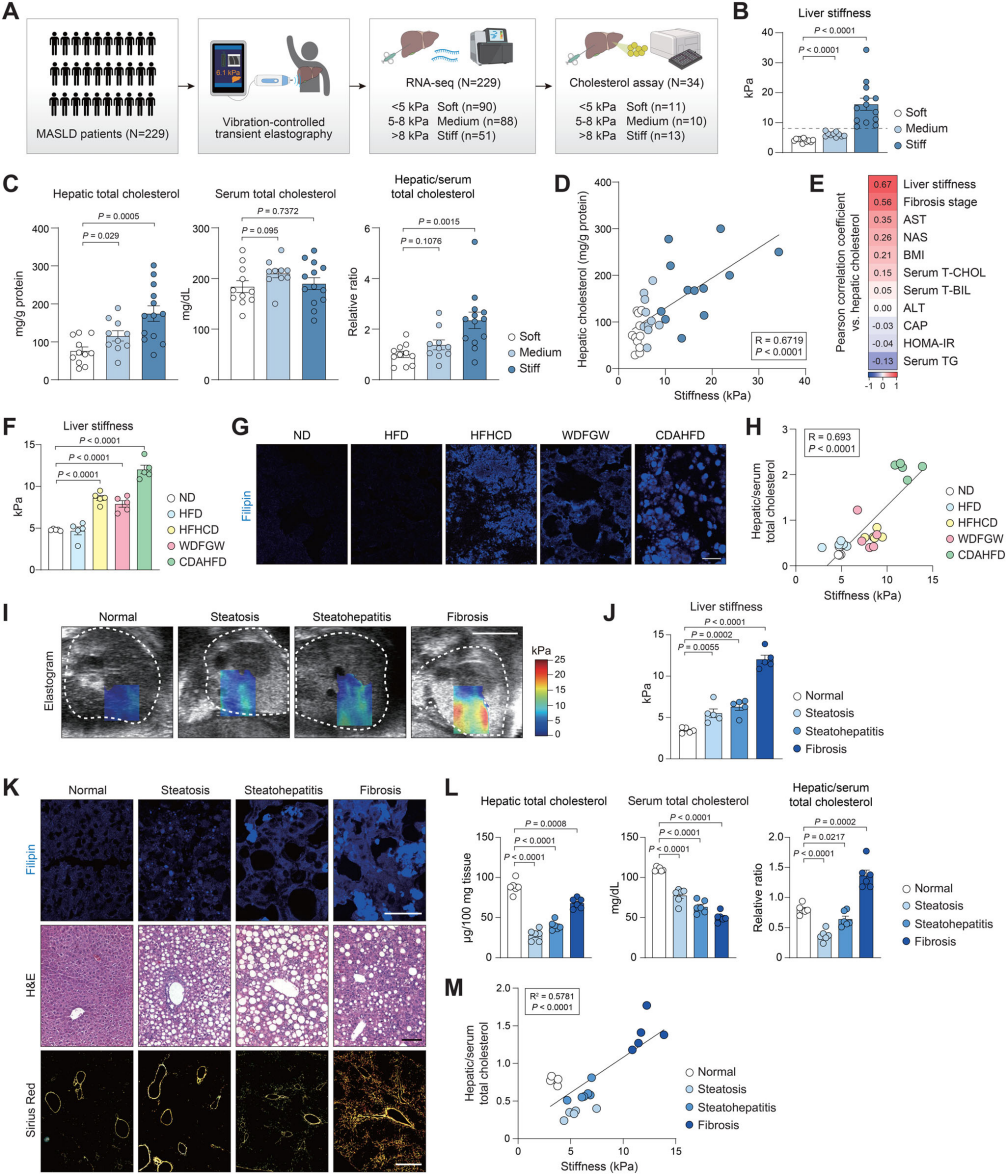
Hepatic cholesterol content correlates with tissue stiffness in MASLD. (A—E) MASLD patients were subjected to transient elastography and routine blood exams. Liver biopsies were taken and analyzed for RNA‐sequencing and hepatic cholesterol quantification. (A) Study design. Patients were categorized into Soft (<5 kPa, *n* = 11), Medium (5–8 kPa, *n* = 10), and Stiff (>8 kPa, *n* = 13) groups based on liver stiffness. (B) Liver stiffness of MASLD patients was categorized into different groups. The dotted line indicates 8 kPa. (C) Total cholesterol levels in the liver and serum. The relative ratio of liver‐to‐serum cholesterol levels was compared. Liver cholesterol levels were normalized to the protein content. (D) Correlation between hepatic cholesterol level and liver stiffness (*n* = 11, 10, and 13). (E) Correlation heatmap of clinical parameters compared with hepatic cholesterol. The numbers represent Pearson correlation coefficients. (F—H) Mice were fed a normal diet (ND) or high‐fat diet (HFD) or high‐fat, high‐cholesterol diet (HFHCD) or western diet with fructose and glucose water (WDFGW), or choline‐deficient, amino acid‐defined, high‐fat diet (CDAHFD) for 12 weeks (*n* = 5). (F) Quantification of liver stiffness measured by SWE. (G) Filipin staining of liver sections. Scale bars, 50 µm. (H) Positive correlation between liver‐to‐serum cholesterol and liver stiffness. (I—M) Mice were fed CDAHFD for 0, 2, 4, and 12 weeks to induce steatosis, steatohepatitis, and fibrosis, respectively (*n* = 6). (I) Representative ultrasound images from mice at indicated times of feeding. Liver stiffness was visualized using shear wave elastography (SWE). Dotted lines indicate livers. Scale bar, 5 mm. (J) Quantification of liver stiffness measured by SWE (*n* = 5). (K) Filipin, H&E, and Sirius Red staining of liver sections from mice fed with CDAHFD. Sirius Red were additionally observed under polarized light for quantification of collagen fibrosis. Scale bars, 50 µm. (L) Total cholesterol levels in the liver and serum. The relative ratio of liver‐to‐serum cholesterol levels was compared. Liver cholesterol levels were normalized to the tissue weight. (M) Positive correlation between hepatic cholesterol and liver stiffness in CDAHFD (*n* = 5). Data are presented as the mean ± s.e.m. *p* values across more than two groups were determined by one‐way ANOVA with Bonferroni's multiple comparisons test. *p‐*values for individual groups compared to control were determined by unpaired Student's *t*‐test.

To investigate the relationship between liver mechanics and cholesterol metabolism, we measured liver stiffness using transient elastography in patients with MASLD. From the entire cohort of 229 patients, 34 individuals with available frozen liver tissues were categorized into 3 groups based on liver stiffness (Figure 1A,B; Figure S1). The cutoff value of 8 kPa was adopted based on clinical guidelines for diagnosing fibrosis; therefore, patients with liver stiffness over 8 kPa were classified as the stiff group [20]. The rest were further divided into soft (<5 kPa) and medium (5–8 kPa) groups to delineate gradual differences within the sub‐pathological range. We found that tissue cholesterol levels were significantly higher in stiffer livers (Figure 1C). Surprisingly, patients with stiff livers exhibited a liver‐to‐serum cholesterol ratio more than three times higher than that of patients with soft livers (Figure 1C). There was a robust positive correlation between liver stiffness and hepatic cholesterol levels across all participants (Figure 1D). In addition, among different clinical parameters that were obtained, liver stiffness exhibited the strongest correlation with hepatic cholesterol levels (Figure 1E), surpassing the histological fibrosis stage.

To further investigate the link between liver stiffening and cholesterol accumulation in the liver, we examined multiple diet‐induced mouse models of MASLD, including high‐fat diet, high‐fat high‐cholesterol diet, western diet supplemented with fructose and glucose water, and choline‐deficient, amino acid‐defined high‐fat diet (CDAHFD) (Figure 1F). Consistently, Filipin staining revealed substantial accumulation of free cholesterol exclusively in stiffer livers (Figure 1G). Liver stiffness exhibited a strong positive correlation with the ratio of liver‐to‐serum cholesterol levels across all models (Figure 1H). Next, we utilized a progressive mouse model representing each stage of MASLD by feeding mice with CDAHFD for 0, 2, 4, and 12 weeks to observe the development of steatosis, steatohepatitis, and fibrosis, as validated in previous studies [21, 22]. As anticipated, 12 weeks of feeding was accompanied by a remarkable increase in liver stiffness, whereas relatively minor changes were observed at earlier time points (Figure 1I,J). Consistent with clinical observations, cholesterol accumulation was prominent only in the fibrotic liver but not in the preceding stages (Figure 1K). This contrasted with the formation of lipid droplets, which increased until steatohepatitis development but diminished in fibrosis. Hepatic cholesterol content and the ratio of liver‐to‐serum cholesterol were significantly higher in the fibrotic liver compared to the livers with steatosis or steatohepatitis (Figure 1L), indicating a greater accumulation of cholesterol from the circulation. The positive correlation between liver stiffness and hepatic cholesterol in mice supported these results (Figure 1M).

### 2.2 Matrix Stiffness Determines Intracellular Cholesterol Level in Hepatocytes

**FIGURE 2:**
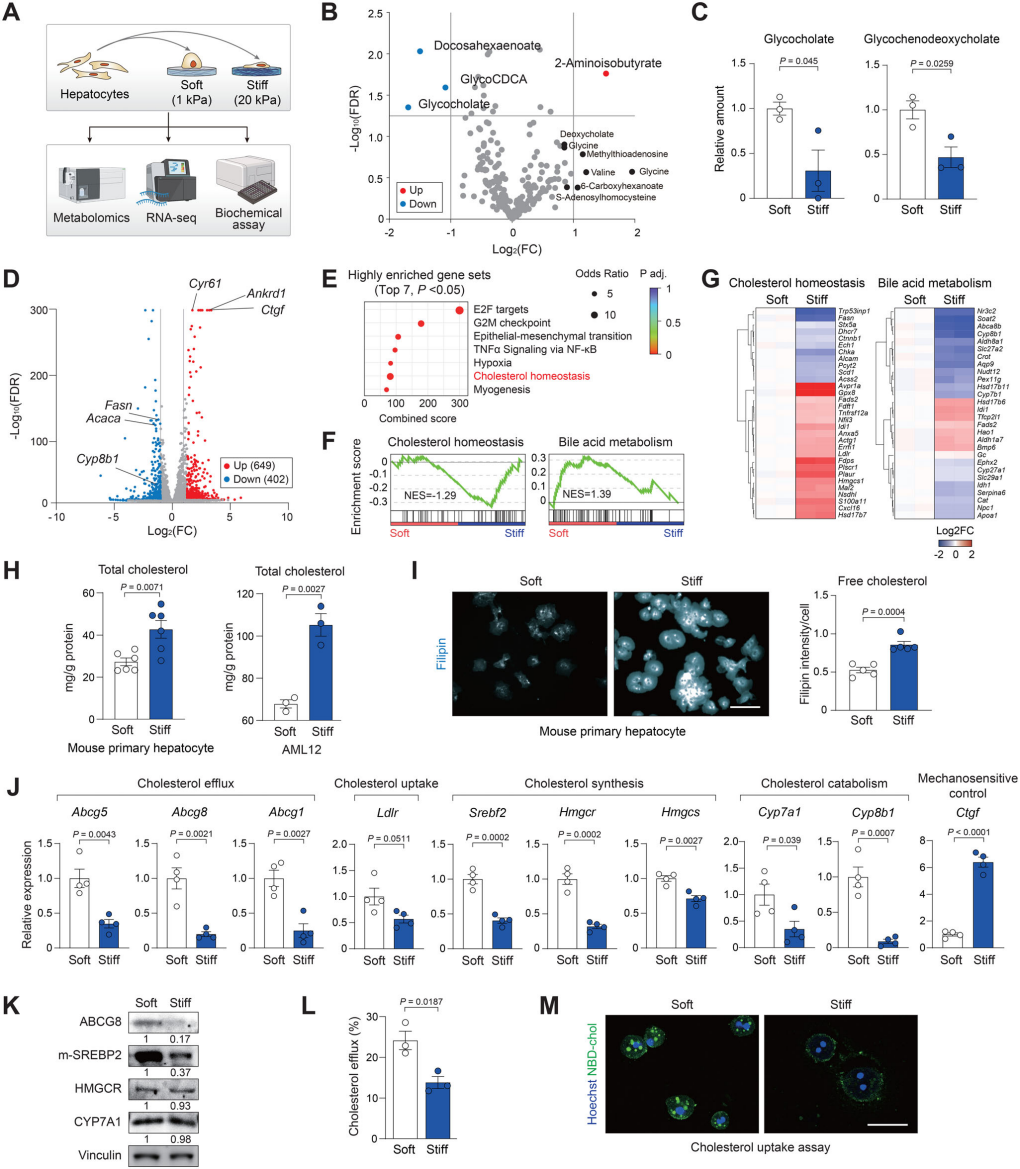
Matrix stiffness determines intracellular cholesterol level in hepatocytes. (A) Study design. Hepatocytes were cultured on soft (1 kPa) or stiff (20 kPa) polyacrylamide hydrogel coated with collagen. After 24 h, metabolites, RNA‐seq, and biochemical assay were analyzed. (B and C) AML12 cells were cultured for 24 h on soft or stiff polyacrylamide hydrogel coated with collagen. (B) Volcano plot of the intracellular metabolite profile (*n* = 3). Significantly altered metabolites are labeled. Colored dots indicate upregulated (red) or downregulated (blue) metabolites. (C) Relative level of downregulated metabolites on stiff hydrogel (*n* = 3). (D–G) Mouse primary hepatocytes were cultured for 24 h on soft or stiff hydrogel and subjected to analyses. (D) Volcano plot of RNA‐seq data showing differentially expressed genes (*n* = 2). Genes involved in cholesterol metabolism and YAP/TAZ target genes are labeled. Colored dots indicate increased (red) or decreased (blue) genes. (E) Hallmark gene set enrichment analysis showing the top 7 significantly altered pathways from RNA‐seq data. (F) GSEA on cholesterol homeostasis and bile acid metabolism from the differentially expressed genes. (G) Heatmap of differentially expressed genes that are related to cholesterol and bile metabolism in hepatocytes. (H) Total cholesterol levels were measured in mouse primary hepatocytes (*n* = 6) and AML12 (*n* = 3). (I) Filipin staining images and quantification of free cholesterol intensity of primary hepatocytes. Same number of cells were seeded. Scale bars, 50 µm. (J) RT–qPCR analysis of cholesterol metabolism‐related genes and the mechanosensitive control gene (*n* = 4). (K) Immunoblot analysis of rate‐limiting proteins of cholesterol regulation. Numbers below each band indicate the relative densitometric values normalized to Vinculin. (L) Cholesterol efflux (%) was calculated from the ratio of fluorescence intensity in the supernatant to the total fluorescence in the supernatant and cell lysate (*n* = 3). (M) NBD‐Cholesterol uptake assay of primary hepatocytes. Nucleus were stained with Hoechst 33342 (blue) and intracellular cholesterol (green) by fluorescence. Quantification of relative cholesterol of primary hepatocytes (*n* = 5). Scale bars, 50 µm. Data are presented as the mean ± s.e.m. *p* values were determined by unpaired Student's *t*‐test.

The observations that cholesterol accumulates along with increasing stiffness in the liver prompted us to investigate which cellular metabolic pathways in hepatocytes are affected by mechanoregulatory signaling. While transient elastography is often used to measure tissue stiffness in the clinical setting, atomic force microscopy (AFM) is utilized for cellular‐level resolution. In healthy individuals, liver stiffness measured by AFM ranges from 2.0 kPa in soft areas to 10.9 kPa in collagen‐rich areas, with some patients showing local stiffness exceeding 100 kPa [23]. Similarly, mean stiffness in mouse livers was measured from 1.9 kPa (healthy) to 5.1 kPa (fibrotic), with certain areas reaching up to 50 kPa [24]. To mimic the healthy and fibrotic microenvironment in human liver, hepatocytes were cultured on either soft (1 kPa) or stiff (20 kPa) collagen‐coated hydrogels and subjected to metabolomics, RNA‐seq, and biochemical analyses (Figure 2A). As a result, intracellular levels of bile salts, such as glycine‐conjugated cholic acid and chenodeoxycholic acid salts, were significantly lower in cells on the stiff substrate (Figure 2B,C). Given that bile salts are major products of cholesterol metabolism, these findings prompted us to further investigate the mechanosensitive expression of cholesterol‐associated genes. Transcriptomic profiling of mouse primary hepatocytes revealed downregulation of genes involved in cholesterol metabolism with upregulation of YAP/TAZ target genes on stiff surface, indicative of augmented mechanotransduction (Figure 2D) [25]. Specifically, cholesterol metabolism pathways were among the top 7 significantly affected pathways (Figure 2E). Gene set enrichment analysis (GSEA) further confirmed the high enrichment of cholesterol homeostasis and bile acid metabolism genes (Figure 2F,G; Figure S2). Indeed, we found significantly higher levels of intracellular cholesterol in hepatocytes on the stiff hydrogel through biochemical quantification (Figure 2H). Filipin staining, which labels free cholesterol [26], also showed mechanosensitive accumulation of cholesterol in hepatocytes on a stiff hydrogel (Figure 2I).

Next, we further analyzed the expression of representative genes involved in cholesterol efflux, uptake, biosynthesis, and catabolism. Intriguingly, primary hepatocytes cultured on the stiff hydrogels showed reduced expression of genes across all these pathways (Figure 2J). At the protein level, ABCG8 was prominently decreased, whereas HMG‐CoA reductase (HMGCR) and CYP7A1 remained largely unchanged (Figure 2K). These findings suggest that the suppression of cholesterol biosynthesis and catabolism under stiff conditions is not a causal mechanism but rather a compensatory response to intracellular cholesterol accumulation. Hepatocytes on stiff substrates showed markedly impaired cholesterol efflux as well as significantly reduced uptake of exogenous cholesterol (Figure 2L,M). Although both cholesterol efflux and uptake were reduced on stiff hydrogels, intracellular cholesterol continued to accumulate, indicating that efflux repression predominates, while uptake reduction represents a compensatory adaptation to excess intracellular cholesterol.

### 2.3 LXRα Activity is Controlled by Substrate Stiffness

The stiffness‐mediated accumulation of intracellular cholesterol was unexpected, since some other cell types, such as Ras‐transformed mammary epithelial cells (MCF10ATk1) and immortalized retinal cells (RPE1), reacted in the opposite direction [27]. This discrepancy led us to postulate that hepatocytes possess a unique mechanosensitive regulatory module governing mechanosensitive cholesterol efflux. Among such modulators is LXRα, a nuclear receptor that governs transcription of ATP‐binding cassette transporters that export cellular cholesterol to the extracellular space and enzymes that metabolize cholesterol [17].

**FIGURE 3:**
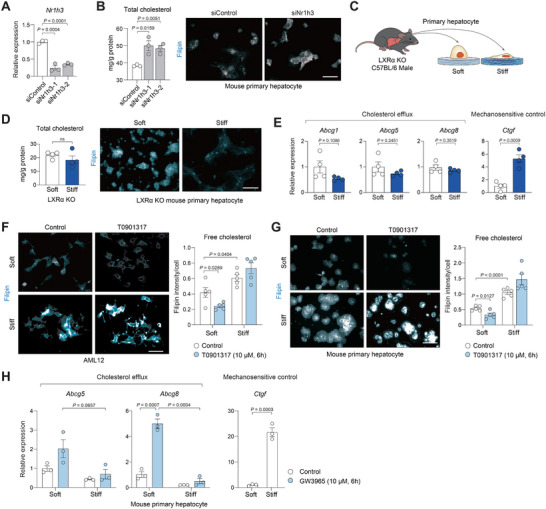
LXRα activity is controlled by matrix stiffness. (A and B) Mouse primary hepatocytes were transfected with siControl or siNr1h3 (LXRα) for 24 h. (A) RT–qPCR analysis of *Nr1h3* expression (*n* = 3). (B) Total cholesterol quantification and Filipin staining of free cholesterol in mouse primary hepatocyte transfected with control or *Nr1h3* siRNA (*n* = 3). Scale bars, 50 µm. (C—E) Primary hepatocytes isolated from LXRα KO mice were cultured on soft or stiff hydrogel for 24 h. (C) Experimental scheme. (D) Total cholesterol levels and free cholesterol for Filipin staining images in the primary hepatocytes. Scale bar, 50 µm. (E) RT‐qPCR analysis of genes regulating cholesterol efflux and metabolism (*n* = 4). (F) Images and quantification of free cholesterol intensity in AML12. Scale bars, 50 µm. AML12 cells were cultured on soft or stiff hydrogels for 24 h and treated with T0901317 (10 µM) for 6 h. (G) Filipin staining images and quantification of free cholesterol intensity in mouse primary hepatocytes. Mouse primary hepatocytes were cultured on soft or stiff hydrogels for 24 h and treated with T0901317 (10 µM) for 6 h Scale bar, 50 µm. (H) RT‐qPCR analysis of cholesterol efflux‐related genes (*n* = 3). Primary hepatocytes from wild‐type mice were cultured on soft or stiff hydrogels for 24 h and treated with GW3965 (10 µM) for 6 h. Data are presented as the mean ± s.e.m. *p* values were determined by unpaired two‐tailed Student's *t*‐test.

To confirm the functional relevance of LXRα in hepatocytes, LXRα (Nr1h3) expression was silenced using siRNA in mouse primary hepatocytes (Figure 3A). As expected, knockdown of LXRα significantly augmented both total and free cellular cholesterol levels (Figure 3B). To further evaluate the contribution of LXRα, primary hepatocytes from LXRα knockout (KO) mice were cultured on soft or stiff hydrogels (Figure 3C). Unlike wild‐type cells, LXRα‐deficient hepatocytes failed to accumulate cholesterol in response to the stiff matrix (Figure 3D), mirroring the non‐hepatocyte cell lines. Also, changes in the expression of genes critical for cholesterol efflux were not observed in the LXRα‐deficient hepatocytes (Figure 3E), suggesting that LXRα is a critical mediator of the mechanosensitive regulation of cholesterol efflux. To investigate whether LXRα activity is influenced by matrix stiffness, AML12 cells were treated with T0901317, a synthetic ligand of LXRα. On soft hydrogel, T0901317 effectively facilitated cholesterol removal from hepatocytes as indicated by decreased Filipin intensity. However, on stiff hydrogel, it failed to reduce cholesterol levels (Figure 3F). Similar results were also found in mouse primary hepatocytes treated with the LXRα agonist (Figure 3G). Additionally, the induction of cholesterol efflux genes that are required for cholesterol removal was significantly blunted on the stiff substrate, providing a mechanistic explanation for these findings (Figure 3H).

### 2.4 YAP/TAZ are the Mechanosensors for LXRα Regulation

**FIGURE 4:**
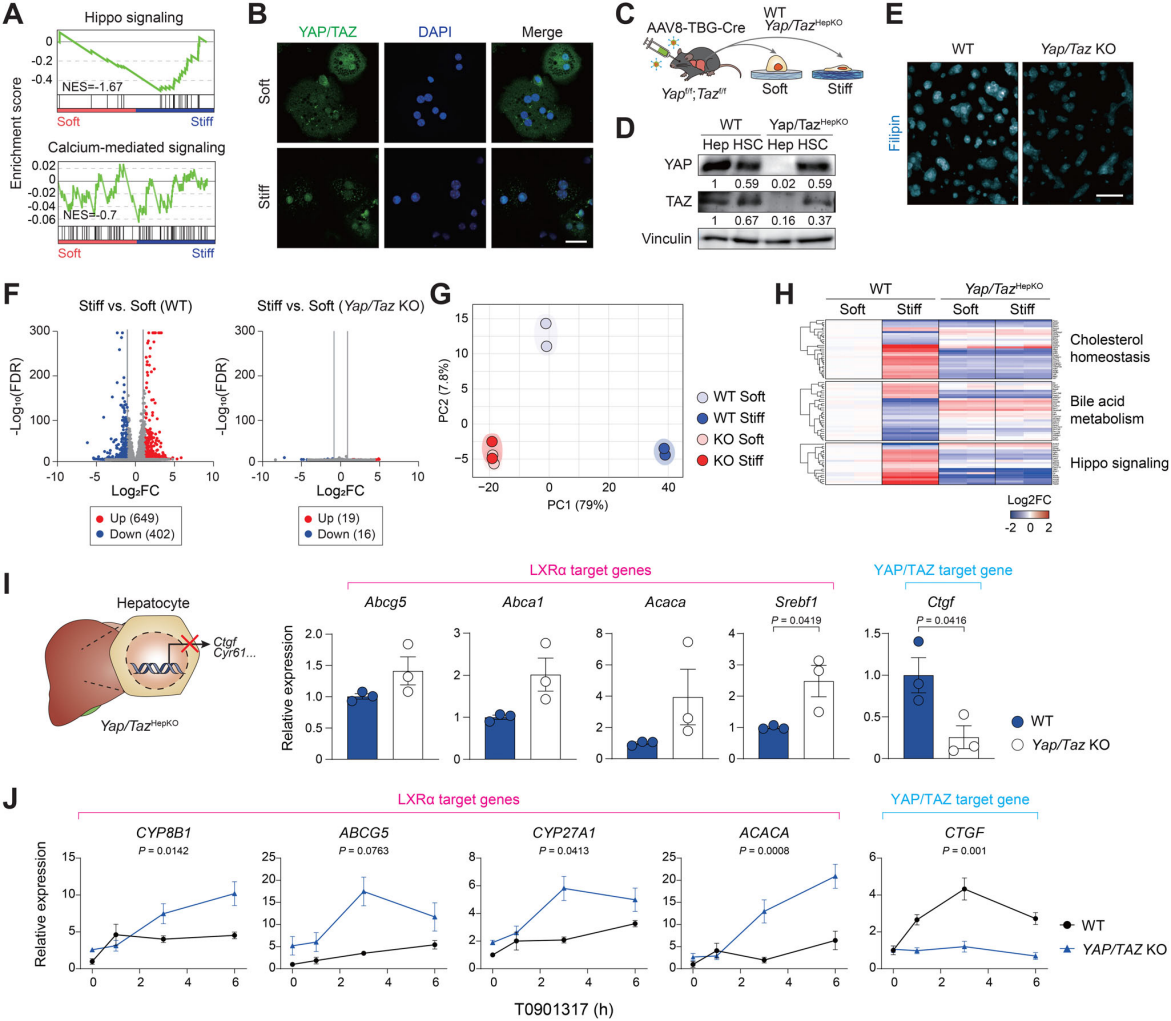
YAP/TAZ mediate mechanotransduction signal to promote cholesterol accumulation in hepatocytes. (A) Gene set enrichment analysis on Hippo signaling and calcium mediated signaling from the differentially expressed genes. (B) Immunofluorescence for YAP/TAZ (green) and DAPI (blue) in mouse primary hepatocytes cultured on soft or stiff hydrogel for 24 h. Scale bar, 50 µm. (C–H), AAV8‐TBG‐Cre or AAV8‐TBG‐Null control virus was injected to *Yap/Taz* ^fl/fl^ mice to yield hepatocyte‐specific *Yap/Taz* knockout (*Yap/Taz* ^HepKO^) or wild‐type (WT) mice, respectively. Each mouse retro‐orbitally received 1 × 10^10^ genomic copies. Primary hepatocytes were isolated after 10 days. (C) Experimental scheme. Primary hepatocytes and hepatic stellate cells from wild‐type and *Yap/Taz* ^HepKO^ were cultured on soft or stiff hydrogel for 24 h. (D) Immunoblot analysis of hepatocytes (Hep) and hepatic stellate cells (HSC) from *Yap/Taz* ^HepKO^ or WT mice. Numbers below each band indicate the relative densitometric values normalized to Vinculin. (E) Filipin staining of primary hepatocytes of the indicated genotype. Scale bar, 50 µm. (F) Volcano plots of RNA‐seq data (*n* = 2). Gene expression from wild‐type (*left*) and *Yap/Taz* KO (*right*) primary hepatocytes cultured on stiff hydrogel vs. soft hydrogel was compared. (G) Principal component analysis (PCA) of metabolic pathways based on gene expression (*n* = 2). (H) Heatmap of differentially expressed genes involved in cholesterol metabolism, bile metabolism, and Hippo signaling pathways (*n* = 2). (I) RT‐qPCR analysis of LXRα target genes in wild‐type and *Yap/Taz* KO hepatocytes (*n* = 3). The data represent the mean ± s.e.m. *p* values were determined by an unpaired two‐tailed Student's *t*‐test. (J) RT‐qPCR analysis for LXRα activity in wild‐type and *YAP/TAZ* KO HEK293 cells (*n* = 3). Cells were treated with T0901317 (10 µM) for the indicated times. Statistical significance was determined by two‐way ANOVA, and *p*‐values indicate the interaction between genotype and time.

To identify the dominant mechanotransduction pathway in hepatocytes, GSEA was compared from the RNA‐seq data of hepatocytes cultured on soft or stiff hydrogels. As a result, the Hippo signaling pathway showed a significant enrichment, whereas the calcium‐mediated signaling pathway associated with other regulators, such as PIEZO1 exhibited only marginal change (Figure 4A). We confirmed that primary hepatocytes were able to induce nuclear localization of YAP/TAZ by culturing on a stiff hydrogel (Figure 4B). These results suggested that hepatocytes primarily sense matrix stiffness through the YAP/TAZ axis.

To determine whether substrate stiffness modulates LXRα activity in a YAP/TAZ‐dependent manner, we utilized AAV8‐TBG‐Cre virus to delete Yap/Taz in hepatocytes of *Yap* ^fl/fl^;*Taz* ^fl/fl^ mice, with AAV8‐TBG‐Null serving as the wild‐type control (Figure 4C). Ten days post injection, successful deletion of Yap/Taz in hepatocytes was confirmed by immunoblotting (Figure 4D). Filipin staining revealed that cholesterol efflux was significantly increased in the absence of Yap/Taz (Figure 4E). Next, RNA‐seq was conducted on wild‐type and Yap/Taz KO primary hepatocytes under different stiffness conditions (Figure 4F). Strikingly, the principal component analysis (PCA) of gene expression revealed that stiffness‐induced gene alternation was significantly abrogated in the absence of Yap/Taz, highlighting that hepatocytes rely heavily on Yap/Taz for mechanotransduction signaling (Figure 4G). Specifically, the transcriptional regulation of mechanosensitive genes associated with cholesterol homeostasis, bile metabolism, and the Hippo signaling pathway was also dependent on the presence of Yap/Taz (Figure 4H; Figure S3). Additionally, RT‐qPCR analysis confirmed that the expression of cholesterol exporters such as *Abca1* and *Abcg5* was modestly increased in Yap/Taz KO hepatocytes (Figure 4I). In YAP/TAZ KO HEK293 cells established by CRISPR‐Cas9, induction of LXRα target genes by T0901317 treatment was dramatically sensitized (Figure 4J), indicating a basal repressive role of YAP/TAZ.

### 2.5 LXRα Activity is Regulated by the Hippo Pathway

**FIGURE 5:**
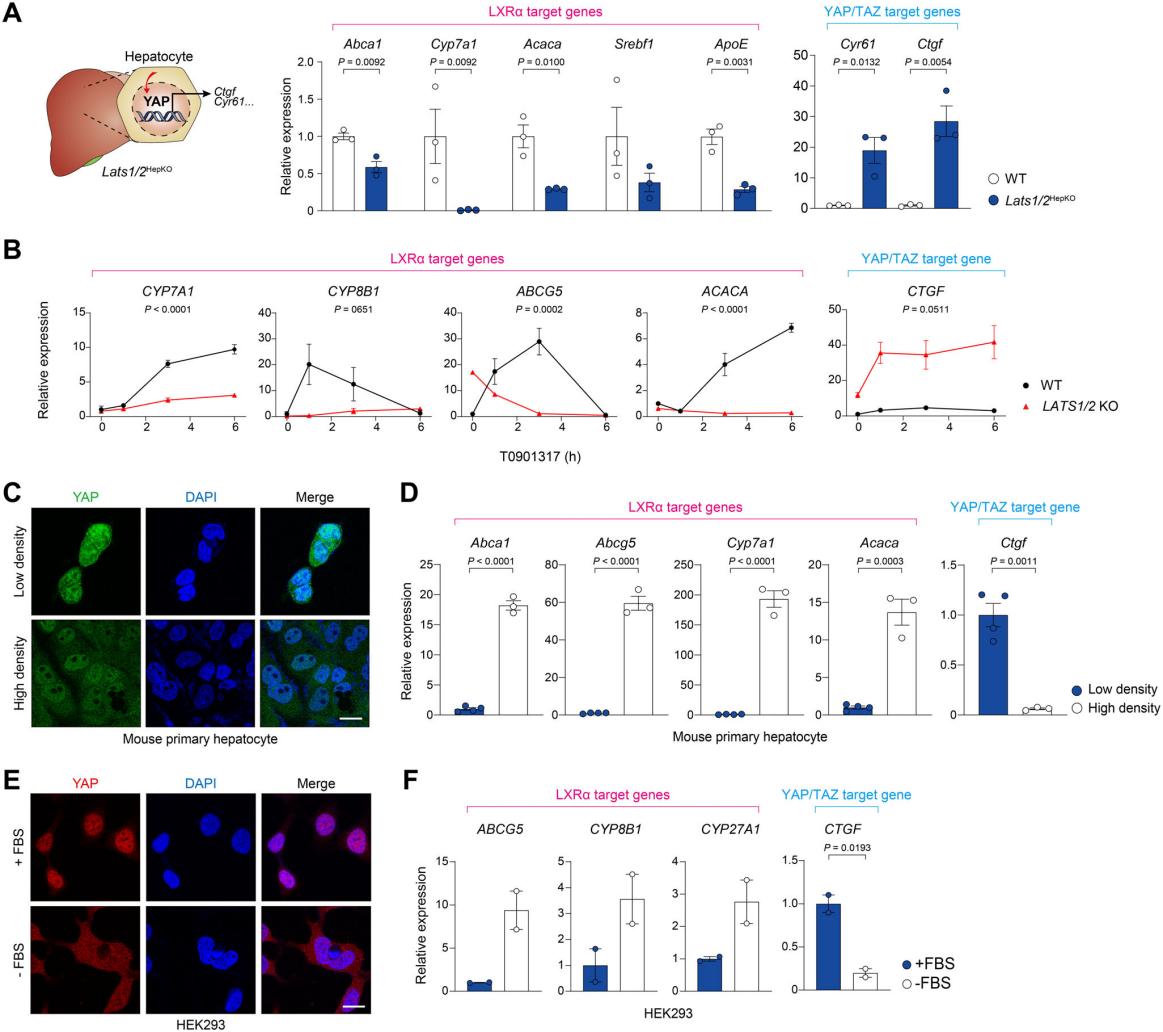
YAP/TAZ activity regulates LXRα signaling and cholesterol metabolism. (A) RT‐qPCR analysis of LXRα target genes involved in cholesterol metabolism (*n* = 3). Bulk livers from *Lats1/2* ^HepKO^ or WT mice were analyzed. (B) RT‐qPCR analysis of LXRα target gene induction in wild‐type and *LATS1/2* KO HEK293 cells (*n* = 2). Cells were serum‐starved for 15 h and treated with T0901317 (100 µM) for the indicated times. Statistical significance was determined by two‐way ANOVA, and *p*‐values indicate the interaction between genotype and time. (C and D) Mouse primary hepatocytes cultured at low (30% confluence) or high (100% confluence) density for 24 h. (C) Immunofluorescence for YAP/TAZ (green) and DAPI (blue). (D) Effect of cell density on LXRα activity (*n* = 4 or 3). RT‐qPCR analysis of LXRα target gene induction is shown. (E and F) HEK293 cells with serum‐starved for 15 h. (E) Immunofluorescence for YAP/TAZ (red) and DAPI (blue). (F) Effect of serum starvation on LXRα activity (*n* = 2). RT‐qPCR analysis of LXRα target gene induction is shown. Data are presented as the mean ± s.e.m. *p*‐values were determined by an unpaired two‐tailed Student's *t*‐test. ns, not significant.

In non‐regenerating adult liver, YAP/TAZ are dominantly repressed by the Hippo pathway kinases Large Tumor Suppressor 1 and 2 (LATS1/2), contributing to the maintenance of organ size [28, 29]. To assess if YAP/TAZ‐mediated repression of LXRα is under control of canonical Hippo pathway signaling, we analyzed livers from hepatocyte‐specific *Lats1/2* KO mice, in which YAP/TAZ are constitutively active [30]. In the *Lats1/2* KO liver, LXRα target gene expression was significantly lower, whereas Yap/Taz target genes (e.g., *Cyr61* and *Ctgf*) were higher when compared to the wild‐type liver (Figure 5A). To further understand whether YAP/TAZ activation impairs transcriptional activity of LXRα, *LATS1/2* KO HEK293 cells were treated with T0901317. RT‐qPCR analysis confirmed the suppressive role of constitutively activated YAP/TAZ on LXRα, as T0901317 failed to induce LXRα target gene expression in the *LATS1/2* KO cells, in contrast to the amplified *CTGF* transcription (Figure 5B).

LATS1/2 are regulated by a variety of extrinsic biophysical and biochemical cues through upstream Hippo pathway kinases [31]. We sought to determine whether Hippo pathway modulation universally controls LXRα activity, irrespective of the specific upstream stimuli. We confirmed that high cell density induced cytoplasmic relocation of Yap/Taz in primary hepatocytes (Figure 5C). High cell density, a condition known to reduce Yap/Taz activity, dramatically increased LXRα target gene transcription and decreased Yap/Taz target gene expression in mouse primary hepatocytes (Figure 5D). Similarly, serum deprivation produced a similar effect, confirming that a reduction in YAP/TAZ activity (Figure 5E) commonly leads to derepression of LXRα (Figure 5F). These data suggest a pivotal interplay between YAP/TAZ and LXRα, providing mechanistic insight into how stiff substrate induces cholesterol accumulation in hepatocytes.

### 2.6 YAP/TAZ Inhibit LXRα Independently of Its Transcriptional Activity

**FIGURE 6:**
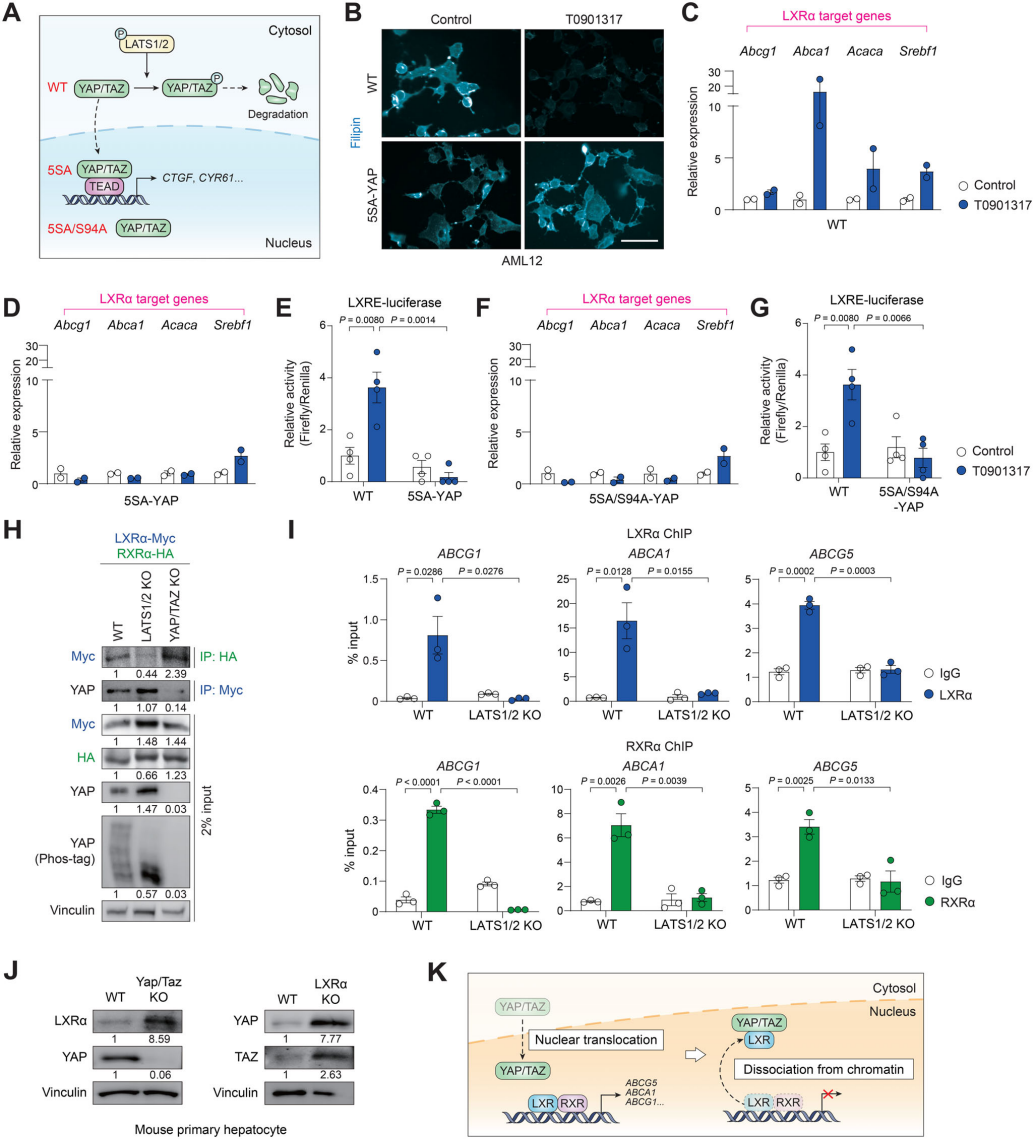
Direct association with YAP inhibits LXRα in the nucleus. (A) A schematic diagram showing the location of YAP with different mutations. In contrast to wild‐type (WT), 5SA mutant is constitutively nuclear due to the lack of phosphorylation by LATS1/2. 5SA/S94A mutant resides in the nucleus but can neither interact with TEAD or induce its canonical target genes. (B) Filipin staining. AML12 cells stably expressing wild‐type YAP or 5SA‐YAP were serum‐starved overnight and treated with T0901317 (10 µM) for 6 h. Scale bar, 50 µm. (C) RT‐qPCR analysis of LXRα target gene induction in cells expressing wild‐type YAP (*n* = 2). AML12 cells were serum‐starved overnight and treated with T0901317 (10 µM) for 6 h. (D) RT‐qPCR analysis of LXRα target gene induction in cells expressing 5SA‐YAP (*n* = 2). Cells were treated as in (C). (E) LXRE reporter activity in wild‐type or 5SA‐YAP cells (*n* = 4). AML12 cells were transfected with a firefly luciferase reporter plasmid containing a synthetic 3xLXRE promoter and treated with T0901317 (10 µM) for 6 h. Renilla luciferase activity was used for normalization. (F) RT‐qPCR analysis of LXRα target gene induction in cells expressing 5SA/S94A‐YAP (*n* = 2). Cells were treated as in (C). (G) LXRE reporter activity in 5SA/S94A‐YAP cells (*n* = 4). Cells were treated as in (e), and the values of wild‐type cells, performed at the same time, were shared. (H) Co‐immunoprecipitation assay of LXRα and RXRα in wild‐type, LATS1/2 KO, and YAP/TAZ KO cells. HEK293 cells of the indicated genotype were transfected with LXRα‐Myc and RXRα‐HA vectors for 24 h (*n* = 3). Cell lysates were immunoprecipitated with Myc or HA antibodies. YAP phosphorylation was determined by Phos‐tag immunoblotting. Numbers below each band indicate the relative densitometric values normalized to Vinculin. (I) Chromatin immunoprecipitation (ChIP) assay (*n* = 3). Association of LXRα (*top*) or RXRα (*bottom*) to the LXRα‐binding regions on ABCG1, ABCA1, and ABCG5 promoters was analyzed in wild‐type and LATS1/2 KO HEK293 cells. Cells were co‐transfected with LXRα‐Myc and RXRα‐HA for 24 h prior to fixation. For input control, 2% of cross‐linked lysates were used. (J) Immunoblot analysis of primary hepatocytes from wild‐type (WT) mice or Yap/Taz KO or LXRα KO. Numbers below each band indicate the relative densitometric values normalized to Vinculin. (K) A schematic diagram showing the role of YAP/TAZ‐mediated inhibition of LXR. Data are presented as the mean ± s.e.m. *p* values were determined by an unpaired two‐tailed Student's *t*‐test.

The Hippo pathway controls YAP/TAZ activity through their nuclear localization, which is tightly prevented by LATS1/2‐mediated phosphorylation on multiple serine residues (Figure 6A) [31]. We generated AML12 cells stably expressing 5SA‐YAP, a constitutively nuclear mutant YAP with all of the LATS1/2 phosphorylation sites mutated to alanine. As expected, cells with 5SA‐YAP efficiently prevented cholesterol efflux facilitated by LXRα in T0901317‐treated cells (Figure 6B). In wild‐type cells, the expression of LXRα target genes such as *Abcg1, Abca1, Acaca*, and *Srebf1* increased after T0901317 treatment (Figure 6C). However, in cells expressing 5SA‐YAP, the induction of these genes was significantly repressed (Figure 6D). Reporter gene assay confirmed that LXR‐responsive element (LXRE)‐dependent promoter activity is repressed when YAP is present in the nucleus (Figure 6E). We thus tested whether YAP/TAZ inhibit LXRα in coordination with their transcriptional partner TEAD. Given that Ser 94 is indispensable for TEAD binding [32], we introduced a 5SA/S94A mutant YAP, which constitutively resides in the nucleus but cannot form YAP‐TEAD heterodimer for transcriptional activation. Unexpectedly, 5SA/S94A‐YAP still retained suppressive activity on LXRα (Figure 6F,G), suggesting that YAP/TAZ may directly interact with LXRα activity within the nucleus independently of their role as transcriptional coactivators.

### 2.7 YAP/TAZ Abolish LXRα‐RXRα Heterodimerization and DNA Binding

To further understand how YAP/TAZ interferes with LXRα activity, we investigated the physical association between LXRα and its indispensable heterodimer partner, Retinoid X receptor alpha (RXRα). In wild‐type cells, LXRα co‐immunoprecipitated with RXRα as expected. However, this interaction was abolished in *LATS1/2* KO cells (Figure 6H). Conversely, *YAP/TAZ* KO cells exhibited stronger heterodimer binding compared to wild‐type cells, indicating that YAP/TAZ act as native inhibitor of the complex formation in the nucleus. Notably, YAP also co‐immunoprecipitated with LXRα in *LATS1/2* KO cells, raising the possibility that YAP competes with RXRα for LXRα binding.

Heterodimerization with RXRα significantly increases the DNA binding affinity of LXRα [33], which is crucial for the effective regulation of target gene expression. Chromatin immunoprecipitation (ChIP) using an anti‐LXRα antibody revealed that LXRα binding to LXREs in the promoters of target genes *ABCG1, ABCG5*, and *ABCA1*, was abolished in *LATS1/2* KO cells (Figure 6I). This suggests that YAP/TAZ‐mediated dissociation from RXRα leads to impaired DNA binding of LXRα. Additional ChIP experiment using an anti‐RXRα antibody showed that YAP/TAZ also dissociates RXRα from the promoter regions of LXRα target genes. Of note, LXRα protein levels were significantly higher in *Yap/Taz* KO hepatocytes, suggesting that Yap/Taz may also promote destabilization of LXRα (Figure 6J). On the other hand, the expression of YAP and TAZ was higher in LXRα KO hepatocytes, demonstrating a mutually suppressive inhibitory regulation between these factors. These findings reveal a novel mechanism by which YAP/TAZ activity disrupts LXRα‐RXRα heterodimerization, impairs LXRα DNA binding, and further promotes its turnover (Figure 6K).

### 2.8 Ablation of Yap/Taz in Hepatocytes Ameliorates Cholesterol‐Induced Liver Fibrosis

**FIGURE 7:**
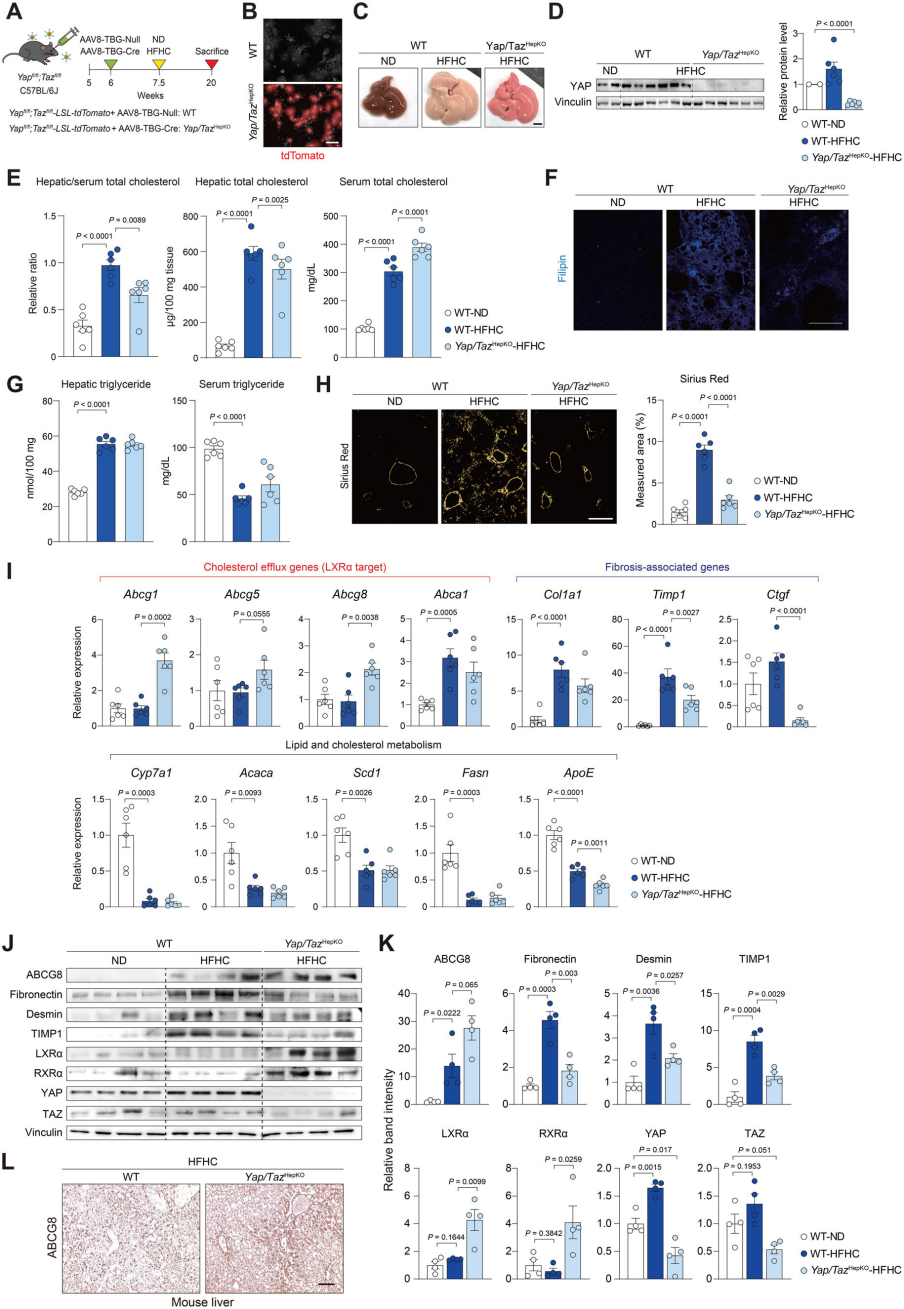
Yap/Taz ablation prevents cholesterol accumulation in the liver and ameliorates cholesterol‐induced liver fibrosis. (A–L) AAV8‐TBG‐Cre or AAV8‐TBG‐Null control virus was injected into Yap/Taz^fl/fl^ mice to yield hepatocyte‐specific *Yap/Taz* knockout (*Yap/Taz* ^HepKO^) or wild‐type (WT) mice, respectively. The mice were then fed with a normal diet (ND) or a high‐fat, high‐cholesterol diet (HFHC) for 12 weeks (*n* = 6). (A) Experimental scheme. (B) Fluorescence images for tdTomato expression in primary hepatocytes. Scale bar, 50 µm. (C) Gross appearance of mouse liver. Scale bar, 5 mm. (D) Immunoblotting for YAP to validate complete gene deletion in mouse liver and quantification of protein levels (*n* = 2 or 6). (E) Total cholesterol levels in the liver and serum. The relative ratio of liver‐to‐serum cholesterol levels was compared. Liver cholesterol levels were normalized to the tissue weight. (F) Representative images of Filipin staining for cholesterol. Scale bar, 50 µm. (G) Triglyceride levels in the liver and serum. (H) Sirius Red staining of liver sections. Scale bar, 50 µm (*left*). Sirius Red‐fibrosis collagen area was quantified under polarized light (*right*). (I) RT‐qPCR analysis of genes associated with cholesterol efflux genes (LXR‐target), fibrosis‐associated genes, and lipid and cholesterol metabolism genes. (J) Immunoblotting of YAP, TAZ, LXRα, RXRα, and fibrogenic proteins in mouse liver (*n* = 4). (K) Quantification of protein levels shown in J. **(L)** Immunostaining of human liver sections for ABCG8. Scale bar, 50 µm. Data are presented as the mean ± s.e.m. *P* values were determined by an unpaired two‐tailed Student's *t*‐test.

Based on our findings that YAP/TAZ activation by increased stiffness promotes cholesterol accumulation, we hypothesized that deletion of YAP/TAZ would prevent hepatocyte cholesterol accumulation during liver fibrosis progression. When comparing mice fed with a high‐fat diet (HFD) or a similar diet with added cholesterol (HFHC), only HFHC diet showed prominent induction of liver fibrosis with a significant induction of total and free cholesterol levels accompanying the increase in liver stiffness (Figure 1F–H). Thus, *Yap* ^fl/fl^;*Taz* ^fl/fl^ mice were injected with AAV8‐TBG‐Cre virus to establish hepatocyte‐specific *Yap/Taz* KO mice (*Yap/Taz* ^HepKO^) and subjected to the cholesterol‐induced fibrosis model (Figure 7A). Gene deletion occurred in virtually all hepatocytes after 10 days of virus injection, indicated by Cre‐mediated expression of tdTomato fluorescence (Figure 7B,C). The ablation of YAP expression was also confirmed by immunoblotting (Figure 7D).

After 12 weeks of HFHC diet feeding, biochemical analysis of liver tissues indicated a clearly lower liver‐to‐serum cholesterol ratio in *Yap/Taz* ^HepKO^ mice, suggesting that loss of *Yap/Taz* resulted in derepression of hepatic cholesterol efflux (Figure 7E). Filipin staining showed significantly suppressed hepatic free cholesterol accumulation in *Yap/Taz* ^HepKO^ mice when compared to the wild‐type mice upon HFHC diet feeding. (Figure 7F). On the other hand, triglyceride levels were not affected by deletion of *Yap/Taz* in our models (Figure 7G). This suggests that Yap/Taz primarily affects cholesterol efflux rather than fatty acid storage during fibrosis. Cholesterol‐induced fibrosis progression was also significantly delayed in *Yap/Taz* ^HepKO^ mice, as evidenced by Sirius Red staining (Figure 7H). As expected, expression of LXRα target genes involved in cholesterol efflux was higher in Yap/Taz^HepKO^ (Figure 7I). This was also confirmed by immunoblot and immunohistochemical analyses. (Figure 7J–L). Because hepatic deletion of *Abcg5/8* impairs biliary cholesterol secretion and drives hepatic cholesterol accumulation to liver injury [34], the increased expression of ABCG8 observed in *Yap/Taz* ^HepKO^ mice supports the protective effect of Yap/Taz loss against cholesterol accumulation. To determine whether Yap/Taz is also involved in intrahepatic cholesterol levels without elevated stiffness, we compared the mice on a simple high‐fat diet with no added cholesterol (Figure S4A–C). Interestingly, significant differences in cholesterol metabolism or fibrosis progression between genotypes were not present (Figure S4D–H). These suggest that the YAP/TAZ‐mediated cholesterol regulation is more substantial in stiff livers where mechanotransduction becomes apparent.

### 2.9 Cholesterol‐Associated LXRα Target Genes Inversely Correlate with Liver Stiffness in Patients with MASLD

**FIGURE 8:**
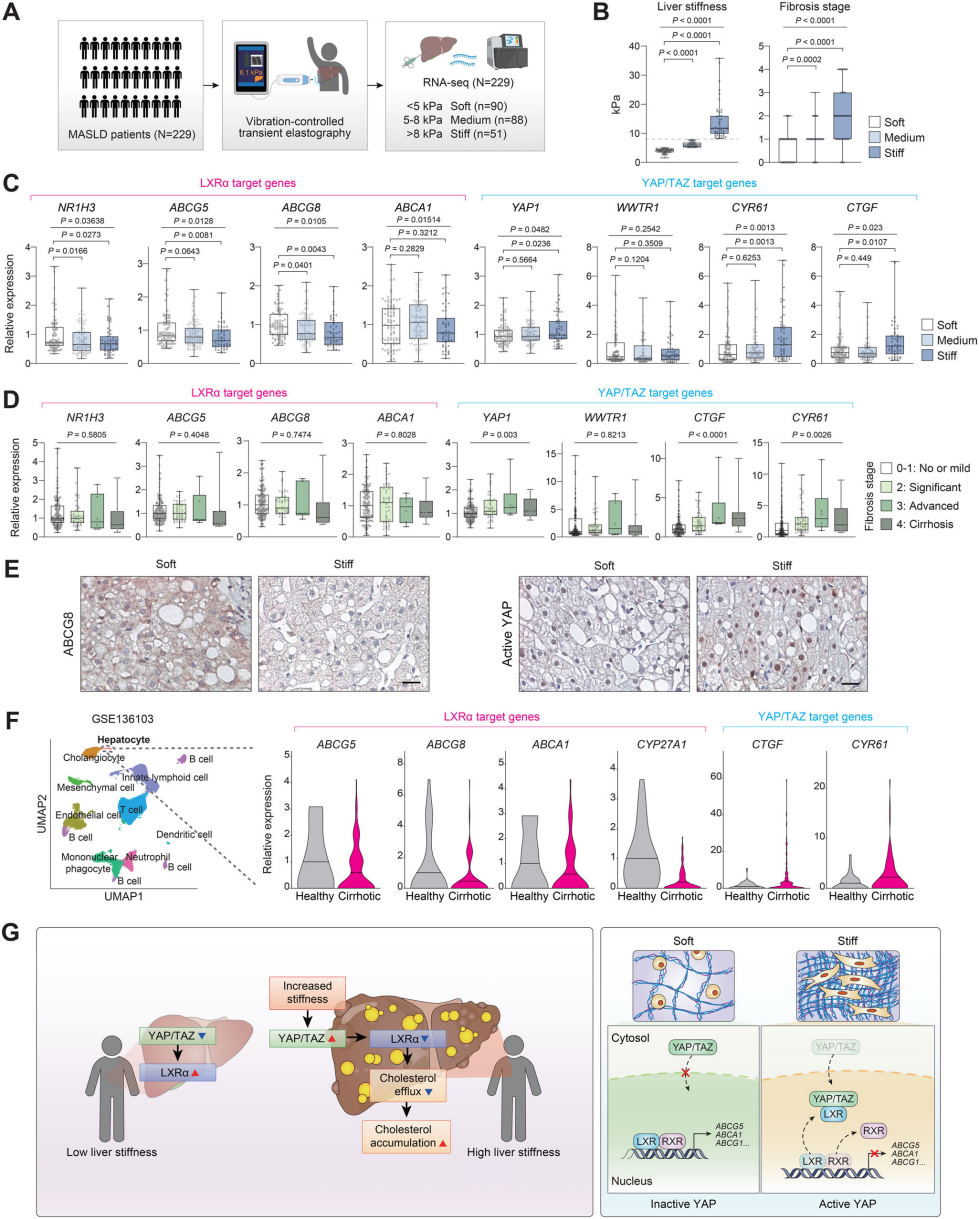
Hepatic LXRα target gene expression inversely correlates with liver stiffness in MASLD patients. (A–E) Individuals with MASLD were subjected to transient elastography (*N* = 229). Liver biopsies were taken and analyzed for RNA‐sequencing. (A) Study design. Patients were categorized into Soft (<5 kPa, *n* = 90), Medium (5–8 kPa, *n* = 88), and Stiff (>8 kPa, *n* = 51) groups based on liver stiffness. (B) Liver stiffness and fibrosis stage of patients in each group. The dotted line indicates 8 kPa. (C) Relative mRNA levels of LXRα and YAP/TAZ target genes from RNA‐seq in each stiffness group. (D) Relative mRNA levels of LXRα and YAP/TAZ target genes in patients grouped for fibrosis stage (*n* = 168, 45, 6, and 10). (E) Immunostaining of human liver sections for ABCG8 and active YAP. Scale bar, 100 µm. (F) Single‐cell RNA‐seq analysis of cirrhotic and healthy human livers (*n* = 5 each). From clusters of single cells by cell lineage, hepatocytes were analyzed for LXRα and YAP/TAZ target gene expression. The data were extracted from GSE136103. (G) A proposed model for the current study. Stiff livers from MASLD patients show elevated cholesterol accumulation, driven by YAP/TAZ‐mediated LXRα repression. In the nucleus, YAP/TAZ disrupts LXRα‐RXRα dimerization. Data are presented as the mean ± s.e.m. *p*‐values across more than two groups were determined by one‐way ANOVA with Bonferroni's multiple comparisons test. *p‐*values for individual groups compared to the control were determined by an unpaired Student's *t*‐test.

To expand our findings on the mechanosensitive regulation of YAP/TAZ and LXRα in clinical settings, we analyzed liver tissues from MASLD patients with varying degrees of liver stiffness. A total of 229 MASLD patients were categorized into soft (<5 kPa), medium (5–8 kPa), and stiff (>8 kPa) subgroups according to liver stiffness as measured by transient elastography (Figure 8A,B). Transcriptomic analysis of liver tissues revealed that transcript levels of LXRα target genes associated with cholesterol efflux were significantly diminished in medium and/or stiff livers when compared to soft livers (Figure 8C). Conversely, YAP/TAZ target genes were significantly upregulated with increasing stiffness. The inverse correlation between liver stiffness and LXRα target gene expression confirmed our findings that LXRα is mechanically regulated in the liver. Intriguingly, this expression pattern could not be observed when patients are grouped according to fibrosis stages (Figure 8D), suggesting that liver stiffness may serve as a valuable diagnostic parameter for estimating the metabolic status, supported by a direct mechanistic link to hepatocyte function. Immunohistochemical analysis confirmed lower ABCG8 expression and higher YAP activity in stiff livers when compared to soft livers (Figure 8E). Additionally, hepatocyte‐directed analysis of single‐cell RNA‐seq data [35] revealed downregulation of LXRα target genes associated with cholesterol efflux, with increased *CTGF* and *CYR61* expression in cirrhotic livers (Figure 8F). Taken together, our results highlight the stiffness‐associated accumulation of hepatic cholesterol in patients with MASLD and reveal the mechanosensitive inhibition of LXRα by YAP/TAZ as the underlying mechanism (Figure 8G).

## 3 Discussion

Increased stiffness is a hallmark of liver fibrosis. However, little is known about how it impacts hepatocyte functions. In this study, we found that matrix stiffness acts as a mechanical cue regulating hepatic cholesterol levels. Our analysis of human MASLD livers demonstrated a strong association between intrahepatic cholesterol and liver stiffness. Hepatocytes cultured on stiff hydrogels spontaneously accumulated more cholesterol. Mechanistically, stiffness‐mediated activation of YAP/TAZ repressed LXRα activity by disrupting its heterodimerization with RXRα. Indeed, *Yap/Taz* ^HepKO^ mice were more resistant to hepatic cholesterol accumulation and fibrosis progression compared with wild‐type mice. Transcriptomic analysis of MASLD patient livers further confirmed an inverse correlation of LXRα target gene expression with liver stiffness and YAP/TAZ target gene expression.

The current dogma for liver fibrosis development in MASLD is that damaged hepatocytes secrete paracrine factors to activate hepatic stellate cells, in which prolonged directional intercellular communication results in excessive accumulation of extracellular matrix (ECM) [36]. Due to this process, the liver undergoes significant remodeling during the progression of liver fibrosis, which involves changes in mechanical properties beyond the physiological range. The current study proposes that changes in liver stiffness are not merely a terminal phenotypic outcome of fiber accumulation. These findings highlight a mechanosensitive feedback loop in which fibrosis‐induced stiffness exacerbates hepatocyte cholesterol accumulation and dysfunction, thereby further accelerating fibrogenesis. While early‐stage disease may respond to interventions targeting steatosis or inflammation, the mechanical remodeling that accompanies fibrosis appears to reinforce disease progression by directly impairing hepatocyte metabolism. This underscores the clinical importance of early therapeutic intervention and suggests that monotherapies directed at hepatocytes may be insufficient once tissue stiffening has taken place.

Based on our results, it can be predicted that responses to treatments such as LXRα modulators may vary depending on liver stiffness. Despite promising preclinical evidence, clinical attempts to control cholesterol levels with LXRα ligands have been unsuccessful due to a lack of efficacy and unwanted adverse effects [37]. Interestingly, similar observations have been made with Resmetirom, another nuclear receptor agonist, whose efficacy appears to diminish in patients with advanced fibrosis. While this has been primarily attributed to irreversible liver damage, our findings raise the possibility that tissue stiffening impairs nuclear receptor signaling, thereby contributing to reduced drug responsiveness in fibrotic livers.

The mechanosensitive pathway we identified represents a potential therapeutic target for MASLD intervention. Current therapeutic approaches focus primarily on metabolic interventions such as GLP‐1 receptor agonists, which have shown promise in reducing liver steatosis and fibrosis markers as measured by transient elastography [38]. However, our findings suggest that targeting the mechanotransduction pathway itself could provide complementary benefits. For instance, targeting integrin‐mediated mechanosignaling pathways has shown promise for treating liver fibrosis [39]. Thus, small‐molecule inhibitors of YAP/TAZ currently being developed for cancer treatment could potentially be repurposed for liver disease [11, 40]. Additionally, targeting downstream effectors of the mechanotransductive pathway, such as specific cholesterol efflux transporters, might provide additional therapeutic approaches.

While our study provides compelling evidence for mechanosensitive regulation of hepatic cholesterol metabolism, several limitations should be acknowledged. First, our in vitro experiments utilized simplified hydrogel systems that may not fully recapitulate the complex mechanical environment of the fibrotic liver. The liver ECM undergoes significant compositional changes during fibrosis progression, beyond just stiffness alterations, which could influence mechanotransduction pathways. In addition, our patient cohort was limited to a single ethnicity, which warrants further validation in diverse populations for generalization. Nonetheless, this study reveals a novel mechanosensitive pathway that creates a vicious cycle where fibrosis‐induced stiffness promotes further cholesterol accumulation and disease progression, providing new insights into MASLD pathophysiology and identifying potential therapeutic targets for intervention. The identification of YAP/TAZ as key hepatic mechanosensors regulating cholesterol homeostasis emphasizes the importance of considering biomechanical factors in liver disease pathogenesis and treatment, potentially leading to more effective strategies to target fibrosis.

## 4 Experimental Sections

### 4.1 Patients

We constructed a prospective cohort from the ongoing Boramae MASLD registry (NCT02206841) [41]. Study participants (*N* = 229) consisted of Korean individuals, aged 19–80, who visited Seoul Metropolitan Government Boramae Medical Center (Table S1). All participants were informed of the study protocol, provided written and signed consent, then after overnight fasting underwent liver biopsy for histological characterization, and vibration‐controlled transient elastography (Fibroscan, Echosens, France) for measuring liver stiffness, respectively, at the same time. Liver tissues were subjected to total RNA isolation for bulk RNA sequencing as previously described elsewhere [42]. Fibrosis staging was assessed according to a 5‐point scale proposed by Brunt and modified by Kleiner et al. [43]. Of the 229 participants with bulk RNA sequencing, 34 subjects with available frozen liver tissues were subjected to hepatic cholesterol quantification assay (Table S2). The study was conducted in accordance with the Declaration of Helsinki and was approved by the Institutional Review Board of Seoul Metropolitan Government Boramae Medical Center (IRB No. 16‐2013‐45).

### 4.2 Animals

LXRα KO mice (#013762; The Jackson Laboratory, ME) were originally established by Dr. David Mangelsdorf (University of Texas, Southwestern). *Yap* ^fl/fl^ mice (#027929; The Jackson Laboratory) and *Taz^f^* ^l/fl^ mice (#032669; The Jackson Laboratory), established by Dr. Eric Olson (University of Texas, Southwestern), were bred to yield *Yap* ^fl/fl^;*Taz* ^fl/fl^;ROSA26‐LSL‐tdTomato mice. For hepatocyte‐specific *Yap/Taz* knockout (*Yap/Taz* ^HepKO^), the *Yap* ^fl/fl^;*Taz* ^fl/fl^ male mice of 7 or 8 weeks old age were intravenously administered with AAV8‐TBG‐Cre virus. As a result, Cre recombinase expression driven by the TBG (thyroxine‐binding globulin) promoter deleted the floxed *Yap* and *Taz* alleles specifically in hepatocytes and simultaneously induced expression of the tdTomato fluorescent reporter. Control mice were given AAV8‐TBG‐Null virus. Hepatocyte‐specific *Lats1/2* KO mice were established in a previous study [30].

For multiple MASLD models, 6‐week‐old male C57BL/6J mice (DBL, Republic of Korea) were randomly grouped. After 1 week of adaptation, mice were fed a chow diet (#38057; Purina, MO), high‐fat diet (#TD88173; Envigo, IN), high‐fat high‐cholesterol diet (#TD02028; Envigo), western diet with fructose, glucose water, or choline‐deficient, L‐amino acid‐defined, high‐fat diet (#A06071302; Research diets, NJ) for 12 weeks [44].

For the progressive MASLD model, 6‐week‐old male C57BL/6J mice were randomly grouped. After 1 week of adaptation, mice were fed choline‐deficient, L‐amino acid‐defined, high‐fat diet for 2 (steatosis), 4 (steatohepatitis), and 12 (fibrosis) weeks, respectively. For the cholesterol‐induced fibrosis experiment, mice were given a high‐fat diet or a high‐fat, high‐cholesterol diet for 12 weeks.

For all experiments, sample sizes were kept minimal based on previous experiences without statistical methods, and animals were randomly allocated to experimental groups with no blinding. Mice were bred and maintained under specific pathogen‐free conditions with a 12‐h dark/12‐h light cycle and controlled temperature and humidity. Animal studies were approved by the Institutional Animal Care and Use Committee of Seoul National University (SNU‐240319‐2, SNU‐220803‐6, SNU‐231006‐2‐1, and SNU‐220902‐1‐1) and were conducted in accordance with institutional guidelines.

### 4.3 Evaluation of Liver Stiffness in Mice

The stiffness of mouse livers was measured using the Vega preclinical ultrasound imaging system (Revvity, MA). Briefly, imaging was performed by anesthetizing mice with vaporized isoflurane (2.5% for induction, 1.5% for maintenance for the duration of imaging), shaving and depilating the abdomen, and orienting in the prone position on the acoustic membrane of the imager with a thin layer of water for coupling. Two‐dimensional shear wave elastograms were acquired per liver at various positions in the left and right lateral lobes below the xiphoid cartilage and quantified per the manufacturer's protocol.

### 4.4 Cell Culture and Treatment

Primary hepatocytes were isolated from the liver of 8‐week‐old male mice by standard two‐step collagenase perfusion and density gradient centrifugation. Isolated hepatocytes were maintained in William's E media (#LM017‐02; Welgene, Republic of Korea) on culture plates coated with 50 µg/mL bovine collagen I (#5005; Advanced Biomatrix, CA). AML12 cells were cultured in DMEM/F12 medium (#LM002‐04; Welgene) with 10% FBS (#F2442; Sigma‐Aldrich, MO) and 1% penicillin/streptomycin (#LS202‐02; Welgene), supplemented with insulin (10 µg/ml), transferrin (5.5 µg/ml), selenium (5 ng/ml), and dexamethasone (40 ng/ml). HEK293 and Huh7 cells were cultured in DMEM (#LM001‐05; Welgene) containing 10% FBS and 1% penicillin/streptomycin. All cells were cultured at 37°C, in a humidified chamber with 5% CO_2_. For LXRα activation, T0901317 (#71810; Cayman Chemical, MI) was used at a concentration of 10 µM for AML12 and primary hepatocytes, and 50 µM for HEK293 cells for 6 h.

### 4.5 Polyacrylamide‐Based Hydrogels with Defined Substrate Stiffness

Hydrogels with different stiffness were prepared as described previously [45] by adjusting the ratio of acrylamide and bis‐acrylamide (#A0418; Gendepot, TX). In brief, polymerized acrylamide hydrogels were treated with 3,4‐dihydroxy‐L‐phenylalanine (#13248; Cayman Chemical) and coated with 50 µg/mL bovine collagen I (#5005; Advanced Biomatrix) to facilitate cell adhesion. The substrates were UV‐sterilized prior to cell culture. Cells were seeded on each substrate overnight.

### 4.6 Metabolomic Analysis

AML12 cells cultured on hydrogels with different stiffnesses were washed with ice‐cold PBS. Intracellular metabolites were extracted using 80% methanol (MeOH) at −80°C, followed by centrifugation at 16 000 g for 20 min. The supernatant was then transferred to glass vials for LC‐MS analysis. The instrumental analysis and data processing were conducted as described elsewhere [46]. For chromatographic separation, 5 µL of each sample was injected into a SeQuant ZIC‐pHILIC LC column (100 × 2.1 mm, 5 µm, 200 Å) coupled with a SeQuant ZIC‐pHILIC guard column (20 × 2.1 mm). The mobile phases consisted of 10 mM ammonium carbonate and 0.05% ammonium hydroxide in water (mobile phase A) and 100% acetonitrile (ACN) (mobile phase B). The column temperature was set to 30°C, and elution was carried out at a flow rate of 0.25 mL/min with the following gradient: 0–13 min, 80% to 20% B; 13–15 min, 20% B (isocratic); 15–15.1 min, 20% to 80% B; followed by a 5‐min postrun at 80% B. Mass spectrometry analysis was performed using a Q Exactive Plus Quadrupole‐Orbitrap (Thermo Fisher Scientific, MA) equipped with a heated electrospray ionization (HESI) source. MS1 scans were conducted in polarity switching mode for nontargeted metabolomics with the following parameter settings: sheath gas 60 arbitrary units, auxiliary gas 25 arbitrary units, sweep gas 2 arbitrary units, spray voltage 3 kV, capillary temperature 320°C, and auxiliary gas heater temperature 370°C. The mass range was 70–1050 m/z, with a resolution of 70 000 and an AGC target of 1 × 10^6^. Data analysis for non‐targeted metabolomics utilized EI Maven v0.12.0. Metabolite identification was conducted by retention time and m/z values of the standard based in‐house library. After the normalization with the median value of the intensities of LC‐MS peaks, the statistical analysis was conducted.

### 4.7 RNA Isolation and Quantitative PCR

Total RNA was isolated using the RNeasy Mini kit (#74106; Qiagen, Germany) or TRIzol reagent (Invitrogen, MA) from cultured cells or mouse livers, respectively. Reverse transcription was done using AccuPower RT premix (Bioneer, Republic of Korea). qRT‐PCR was performed using AccuPower 2X GreenStar RT‐qPCR Master Mix (Bioneer) using the CFX Connect Real‐Time PCR detection system (Bio‐Rad, CA). After polymerase chain reaction amplification, the melt curve of each amplicon was determined to verify its accuracy. All mRNA levels were normalized to the expression of housekeeping genes. Primer sequences used for PCR are available in Table S3.

### 4.8 Transcriptomic Analyses

Primary hepatocytes from wild‐type or *Yap/Taz* ^HepKO^ mice were cultured in hydrogels with different stiffness for 24 h. RNA was prepared using RNeasy Mini kit (Qiagen). cDNA synthesis was performed using dUTP and sequenced on the Illumina platform (paired end 150 bp). Sequencing reads were aligned to the mouse reference genome using HISAT2 (v2.0.5), and gene‐level counts were obtained with featureCounts (v1.5.0‐p3). Differential expression analysis was performed using the DESeq2 R package (v1.50.2) on raw count data, and differentially expressed genes (DEGs) were defined as those with an adjusted *p* value (false discovery rate, FDR) <0.05 and |log_2_ fold change| > 0.5. Normalized expression values were used for downstream visualization and clustering analyses. Heatmaps were generated using the pheatmap (v1.0.13) based on a subset of DEGs corresponding to genes included in the Molecular Signatures Database (MSigDB) Hallmark and Gene Ontology Biological Process (GOBP) gene sets. Pathway enrichment analysis was performed using the clusterProfiler R package (v4.18.4) via over‐representation analysis (ORA) based on Hallmark gene sets, with enrichment significance assessed by highly enriched gene sets and adjusted *P* values. In parallel, Gene Set Enrichment Analysis (GSEA) was carried out using the GSEA desktop application (Broad Institute, v4.3.2) with 1000 gene set permutations, the weighted enrichment statistic, and Ratio_of_Classes as the metric. All gene sets obtained from MSigDB or GOBP databases corresponded to the 2025.1.Mm release. Principal component analysis (PCA) was performed using variance‐stabilized gene expression values generated by DESeq2, and computed with the prcomp function in base R, using all expressed genes.

Single‐cell RNA‐seq data (GSE136103) [35] were retrieved from the Gene Expression Omnibus. This dataset comprises 66 135 liver‐resident cells from human livers (*n* = 5 each). All analyses were performed in R using Seurat (v5.3.1), SeuratObject (v5.2.0), Harmony (v1.2.4), and ggplot2 (v4.0.0). For each sample, count matrices were converted into Seurat objects with SeuratObject. Quality control was performed in Seurat, and cells were retained if they had 500–6000 detected genes, >1000 UMIs, and <25% mitochondrial transcript content. Counts were log‐normalized, and the top 2000 most highly variable genes were identified using Seurat. Batch effects across donors and fractions were corrected using Harmony. Harmony‐corrected embeddings were then used within Seurat to construct a shared nearest‐neighbor graph and to perform graph‐based clustering with the Louvain algorithm at a resolution of 0.6. Two‐dimensional visualization was performed using UMAP based on the Harmony embeddings, and all plots were generated with ggplot2. Cell type annotation was guided based on curated representative marker genes, as also performed by the original study [35] (Figure S5).

### 4.9 Filipin Staining

To visualize intracellular cholesterol accumulation, frozen liver sections and live cultured cells were fixed in 4% paraformaldehyde solution for 1 h at room temperature, followed by quenching with glycine solution for 10 min. Cells were stained with 0.05% solution of Filipin (#F9765; Sigma‐Aldrich) and examined using fluorescence microscopy with a DAPI filter set in PBS.

### 4.10 siRNA‐Mediated Gene Silencing

Mouse primary hepatocytes were transfected with 10 nM of 27‐mer siRNAs targeting Nr1h3 (LXRα) using Lipofectamine RNAiMAX (Invitrogen) according to the manufacturer's protocol. After 24 h, knockdown efficiency was assessed by qRT‐PCR. The sequence of siRNAs is provided in Table S3.

### 4.11 DNA Transfection

Cells were transfected with plasmids using Lipofectamine 3000 (Invitrogen) according to the manufacturer's protocol. For transfection, pQCXIH‐Myc‐YAP, pQCXIH‐Myc‐5SA‐YAP, pCMV‐Flag‐5SA‐YAP, pCMV‐Myc‐hLXRα, pRK7‐HA‐RXRα were used. Experiments were done at least 24 h after transfection.

### 4.12 Western Blot

Gels containing Phos‐tag (Wako Chemicals, Japan) were prepared according to the manufacturer's instructions. Cell or tissue lysates were quantified, separated by SDS‐PAGE, and transferred to nitrocellulose membranes (GE Healthcare, IL) followed by immunoblotting. For immunoprecipitation, wild‐type, *LATS1/2* KO, and *YAP/TAZ* KO HEK293 cells transfected with LXRα and RXRα plasmids using Lipofectamine 3000 (Invitrogen) were lysed with buffer containing 150 mM NaCl, 1 mM EDTA, 0.5% NP‐40, and 10% Glycerol, and phosphatase/protease inhibitor cocktail. The lysates were precleaned by centrifugation and incubation with Protein A beads (#L00273; GenScript, NJ) for 4 h at 4°C with gentle rotation. Lysates were then incubated with the desired antibodies and Protein A beads. Immunoprecipitated proteins were eluted by boiling beads in a denaturing buffer and subjected to immunoblotting using primary antibodies and Clean‐Blot HRP‐conjugated secondary antibody (#21230; Thermo Fisher Scientific). For immunoblotting, proteins of interest were probed with primary antibodies and horseradish peroxidase‐linked secondary antibodies for chemiluminescence detection. Full uncut blots are provided in Figure S6. Antibodies used for immunoblottings are listed in Table S4. Band intensities were quantified using ImageJ (1.54 k) software. The intensity of each target protein band was normalized to Vinculin as a loading control, and the resulting values were further normalized to the control group to determine the relative protein expression levels.

### 4.13 Immunofluorescence

Cells were seeded on coverslips pretreated with poly‐L‐ornithine (#P3655; Sigma‐Aldrich). The cells were fixed with 4% paraformaldehyde for 30 min and permeabilized with 0.1% Triton X‐100. After washing, the cells were stained with an anti‐YAP/TAZ antibody and mounted using Vectashield HardSet with DAPI (#H‐1500‐10; Vector Laboratories, CA). Images were acquired using a confocal microscope (#TCS7; Leica, Germany).

### 4.14 Luciferase Assay

HEK293 cells were co‐transfected with LXRE and Renilla luciferase vectors for 24 h using Lipofectamine 3000 (Invitrogen). Luciferase activity was measured using a luciferase assay reagent (#E1910; Promega, WI). Firefly luciferase intensities were normalized by the respective Renilla luciferase activity in each sample.

### 4.15 Chromatin Immunoprecipitation

Wild‐type and *LATS1/2* KO HEK293 cells were transfected with plasmids expressing LXRα and RXRα using Lipofectamine 3000 (Invitrogen). Cells were then treated with T0901317 for 6 h, and 1% formaldehyde was added for chromatin cross‐linking. Chromatins were immunoprecipitated using SimpleChIP assay kit (#9004; Cell Signaling Technology, MA). Normal rabbit IgG was used as a negative control. One tenth of cross‐linked lysates served as the input control. The primers used in the PCR assays are listed in Table S3.

### 4.16 Biochemical Quantification of Cholesterol

Snap‐frozen human liver biopsies were homogenized in a lysis buffer containing protease inhibitor and quantified for cholesterol using Amplex Red Cholesterol Assay Kit (#A12216; Invitrogen). For normalization, the protein concentration of each lysate was measured by Bradford assay. For mouse livers, 100 mg of tissue was homogenized in 200 µL of chloroform:isopropanol:NP‐40 (7:11:0.1) solution and centrifuged for 10 min. Organic phase was taken and air‐dried at 50°C to remove the chloroform. Dried lipids were then quantified for cholesterol using the Total Cholesterol Assay Kit (#BM‐CHO‐100; BioMax, Republic of Korea). Serum cholesterol in mice was quantified using DRI‐CHEM NX500 biochemical blood analyzer (Fujifilm, Japan).

### 4.17 Cholesterol Uptake and Efflux Assays

For the cholesterol uptake assay, Cholesterol Uptake Assay Kit (#ab236212; Abcam, United Kingdom) was used. Mouse primary hepatocytes were incubated with NBD‐labeled cholesterol in serum‐free medium for 24 and 48 h. Intracellular fluorescence was visualized using a confocal microscope. NBD‐cholesterol was detected in GFP channel. Nuclei were stained with Hoechst 33342 (#H1399; Invitrogen).

Cholesterol efflux was examined using the Cholesterol Efflux Assay Kit (#ab196985; Abcam). Mouse primary hepatocytes were first loaded with a fluorescent cholesterol labeling reagent. After equilibration, the cells were subjected to serum‐free medium containing cholesterol acceptors. Culture medium and cell lysates were respectively collected, and fluorescence intensity was measured using SpectraMax iD3 Multi‐Mode Reader (Molecular Devices, CA). Efflux rate was calculated by dividing medium fluorescence by (medium+cell lysate fluorescence).

### 4.18 Gene Editing

pSpCas9(BB)‐2A‐Puro (#62988; Addgene, MA) and lentiCRISPRv2 (#52961; Addgene) were used for CRISPR‐guided gene knockout. Briefly, guide RNAs were cloned into the empty vector. Cells were then transfected with plasmids, selected for puromycin resistance, and FACS‐sorted for single cells. Expanded single clones were screened by protein immunoblotting and verified by genomic DNA sequencing in a previous study [47]. The sequences for guide RNAs used for gene editing are listed in Table S3.

### 4.19 Statistical Analysis

All statistical analysis was performed using GraphPad Prism, and values are expressed as means ± s.e.m. *p* values across more than two groups were determined by one‐way ANOVA with Bonferroni's multiple comparisons test. *p* values for individual groups compared to the control were determined by an unpaired Student's *t*‐test. Histological quantification of slides stained with Sirius Red was done using FibroSoft.

## Author Contributions

N.Y.L. and J.H.K. contributed to the conceptualization of the project. N.Y.L. performed experiments and data visualization. M.G.C., Y.J.M., S.M.K., Y.P.K., W.K., and B.K.K. contributed by conducting supportive experiments. Y.C. provided mouse samples. H.J.R. performed single‐cell analysis. J.H.K. secured funding, supervised the project, and contributed to project administration. N.Y.L. and J.H.K. wrote the original draft, and W.K. and J.H.K. reviewed and edited the manuscript.

## Conflicts of Interest

The authors declare no conflicts of interest.

## Supporting information




**Supporting File**: advs74494‐sup‐0001‐SuppMat.docx.

## Data Availability

RNA‐sequencing data generated in this study have been deposited to the Gene Expression Omnibus repository (GSE274047).
