# Supplementary material for: Liver Stiffness Directs Intrahepatic Cholesterol Accumulation Through YAP/TAZ in Metabolic Dysfunction‐Associated Steatotic Liver Disease
Source: Adv Sci (Weinh). 2026 Feb 26;13(25):e00379. doi: 10.1002/advs.202500379 (PMC13137846; doi:10.1002/advs.202500379)
Supplement: Supplementary file 1 — Supporting File: advs74494‐sup‐0001‐SuppMat.docx. [file ADVS-13-e00379-s001.docx]

**Liver stiffness directs intrahepatic cholesterol accumulation through YAP/TAZ in metabolic dysfunction-associated steatotic liver disease**

Na Young Lee, Myeung Gi Choi, Ho Jae Ryu, Young Jin Min, Seon Min Kim, Bo Kyung Koo, Yeonseok Chung, Yun Pyo Kang, Won Kim, Ja Hyun Koo

Supplemental Figures 2

Supplemental Tables 10


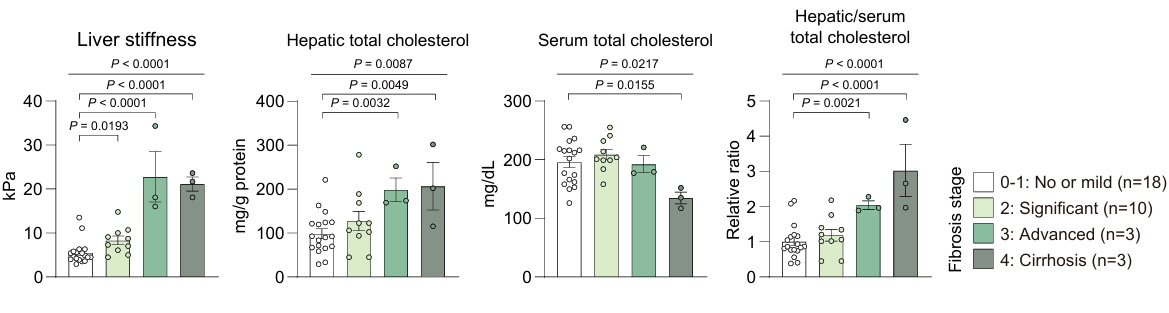
**Supplemental Figures**

**Figure S1. Fibrosis stage was not a significant predictor of intrahepatic cholesterol levels.**

Patients were categorized into F0-1 (No or mild fibrosis, n =18), F2 (Significant fibrosis, n = 10), F3 (Advanced fibrosis, n = 3), and F4 (Cirrhosis, n = 3). Liver stiffness of patients in each group and hepatic cholesterol quantification were analyzed.

**Figure S2. Enlarged heatmap**


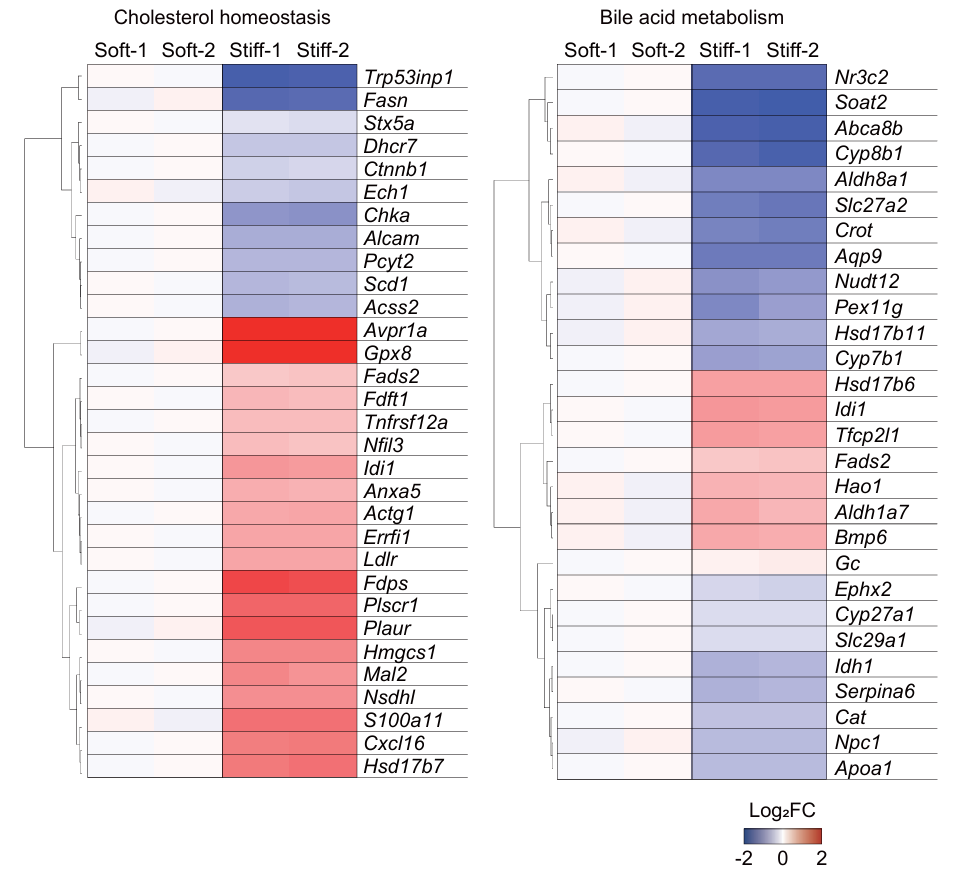


Enlarged heatmap of **Fig 2G**.

**Figure S3. Enlarged heatmap**


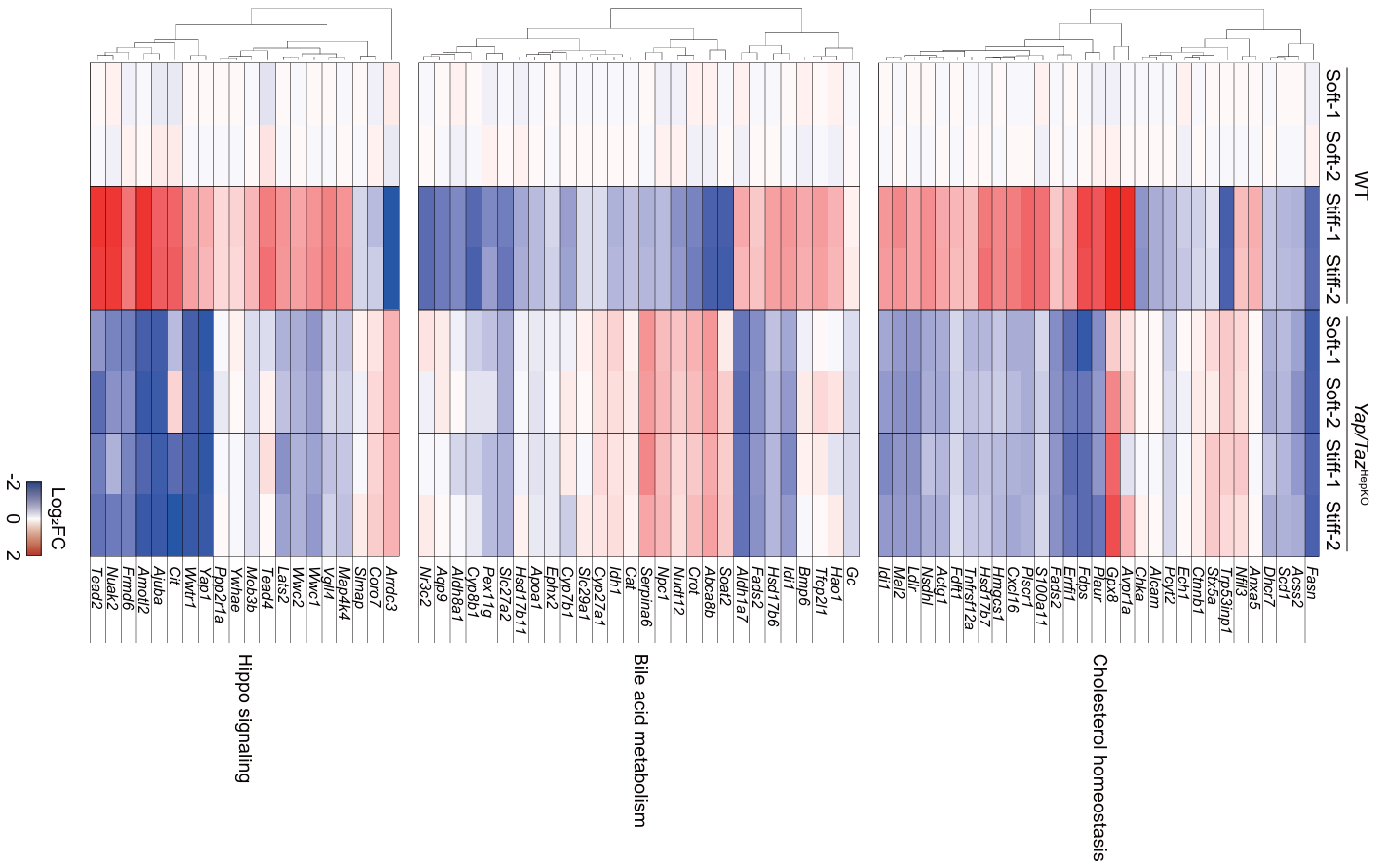


Enlarged heatmap of **Fig 4G**.

**Figure S4. Fibrosis stage was no a significant predictor of intrahepatic cholesterol levels.**

**
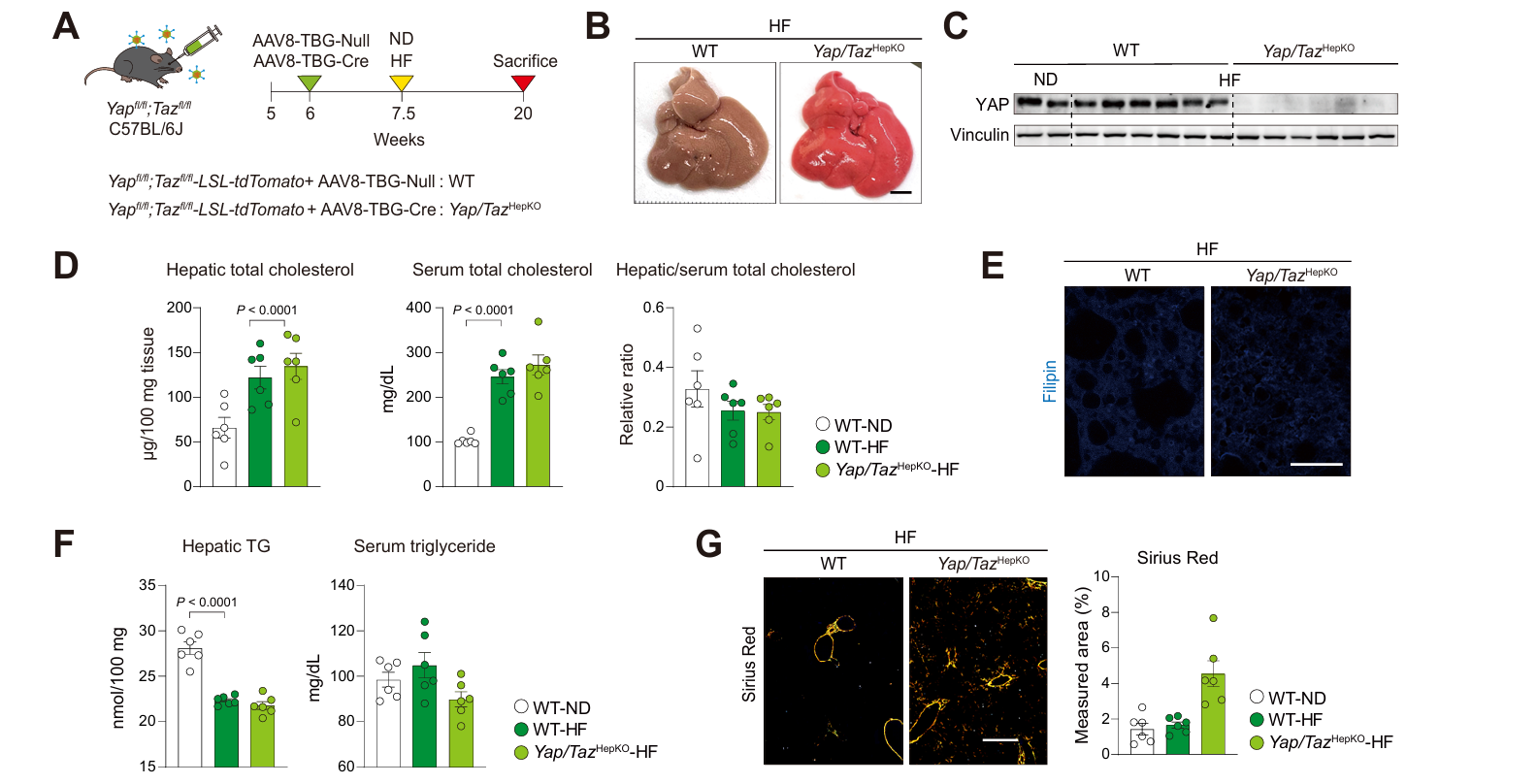
**

**
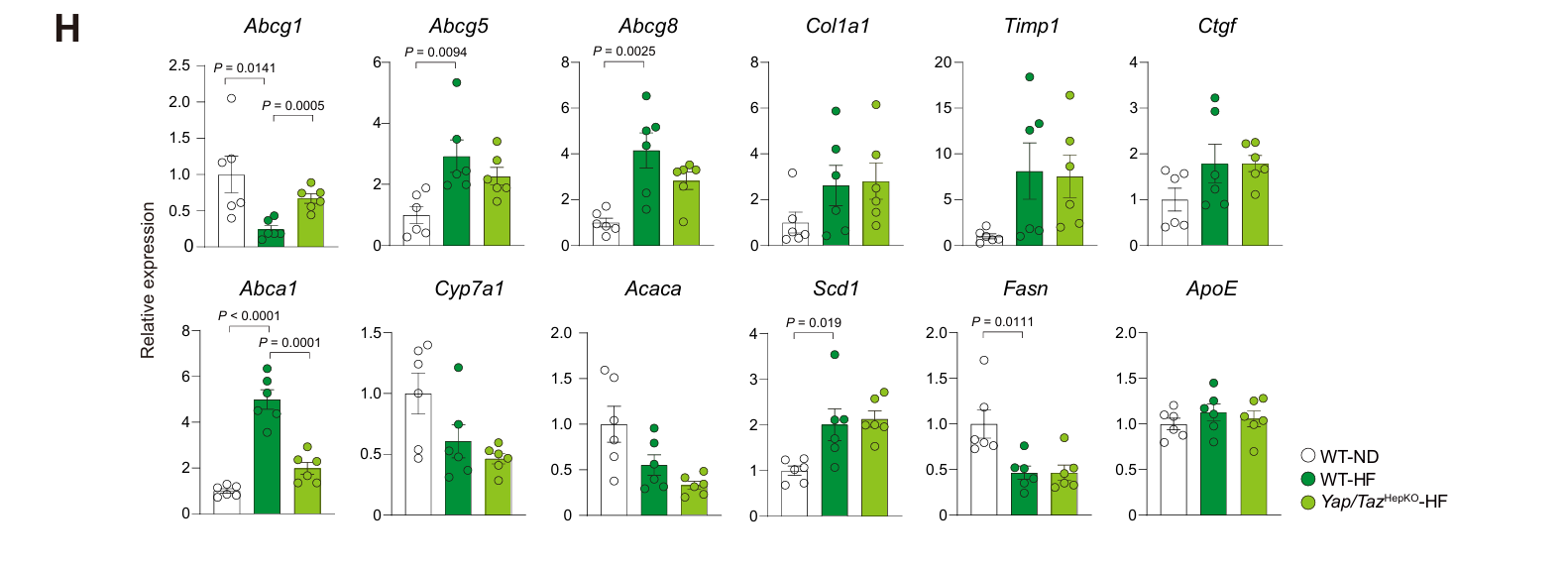
**

**(A – H)** AAV8-TBG-Cre or AAV8-TBG-Null control virus was injected to Yap/Taz^fl/fl^ mice to yield hepatocyte-specific *Yap/Taz* knockout (*Yap/Taz*^HepKO^) or wild-type (WT) mice, respectively. The mice were then fed with a normal diet (ND) or high-fat (HF) diet for 12 weeks (n = 6). **(A)** Experimental scheme. **(B)** Fluorescence images for tdTomato expression in primary hepatocytes. Scale bar, 50 μm. **(C)** Gross appearance of mouse liver. Scale bar, 5 mm. **(D)** Total cholesterol levels in the liver and serum. The relative ratio of liver-to-serum cholesterol levels was compared. Liver cholesterol levels were normalized to the tissue weight. **(E)** Representative images of Filipin staining for cholesterol. Scale bar, 50 μm. **(F),** Triglyceride levels in the liver and serum. **(G),** Sirius Red staining of liver sections. Scale bar, 50 μm (*left*). Sirius Red-fibrosis collagen area was quantified under polarized light (*right*, n = 6 each). **(****H),** RT-qPCR analysis of genes associated with cholesterol efflux genes (LXR-target), fibrosis-associated genes, and lipid and cholesterol metabolism genes.

**Figure S5. UMAP of liver cell types and marker gene expression**

**
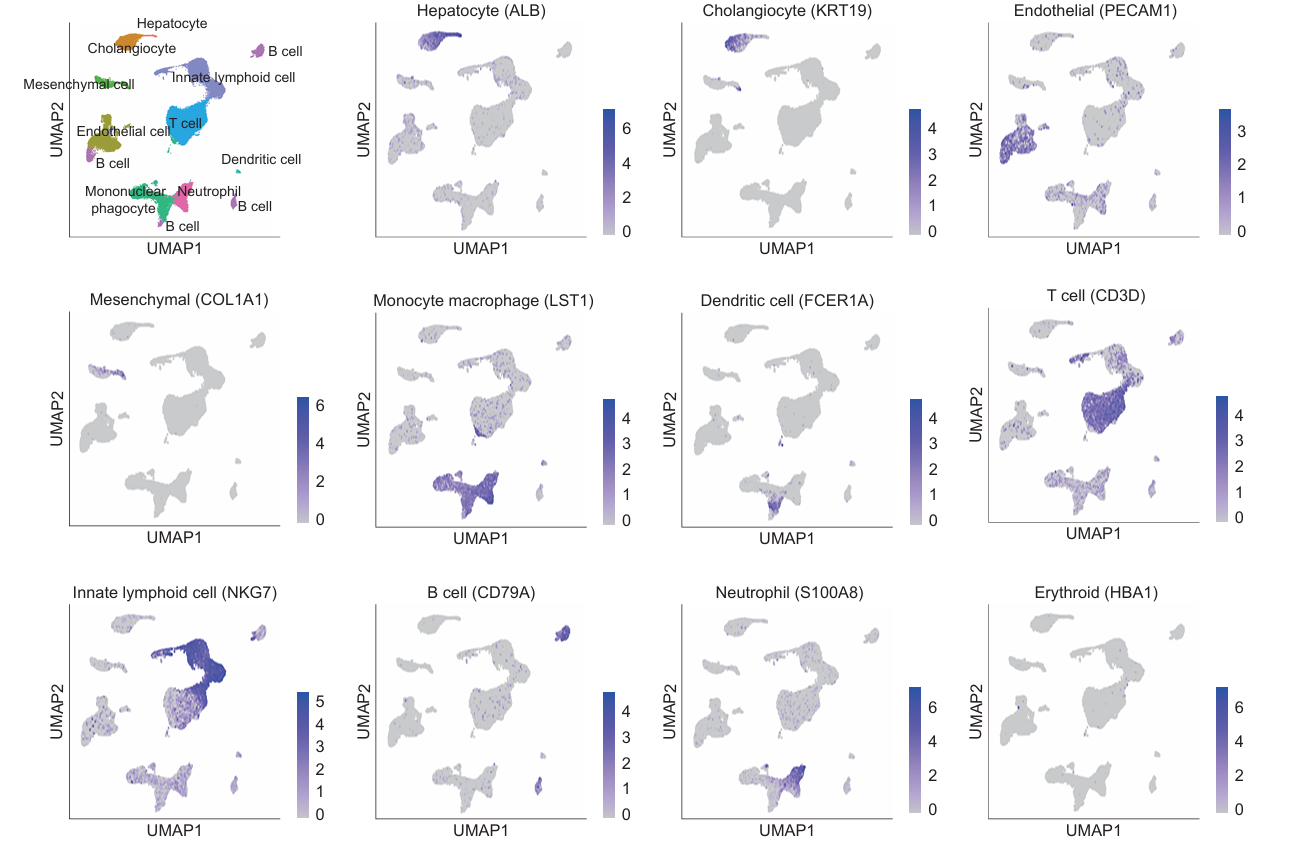
**

UMAP embedding of all liver-resident cells colored by major cell-type annotations. Cells were clustered and annotated as hepatocytes, cholangiocytes, endothelial cells, mesenchymal cells, monocyte/macrophages, dendritic cells, T cells, B cells, innate lymphoid cells, neutrophils, and erythroid cells. Color scales indicate normalized expression levels of each gene on the shared UMAP1–UMAP2 embedding, with gray indicating low or no expression.

**Figure S6. Uncut blots**

**
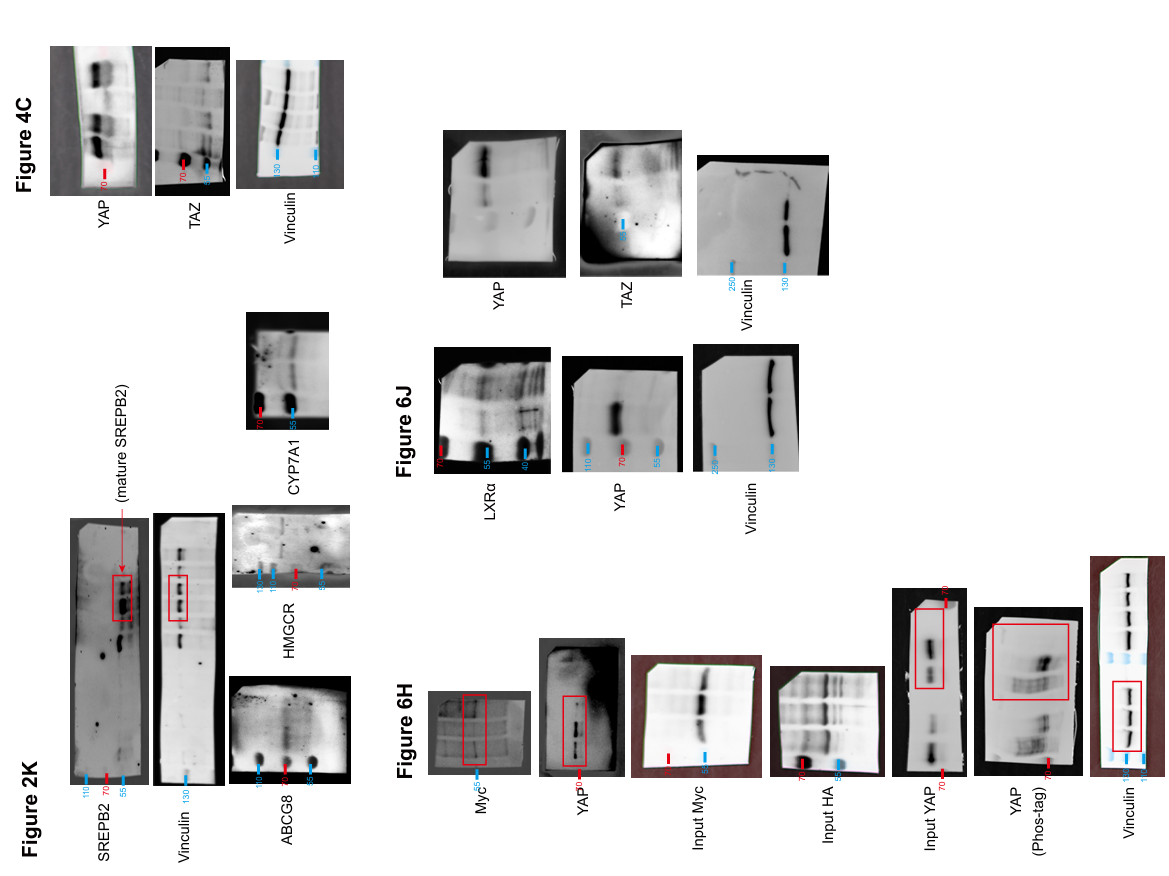
**

*
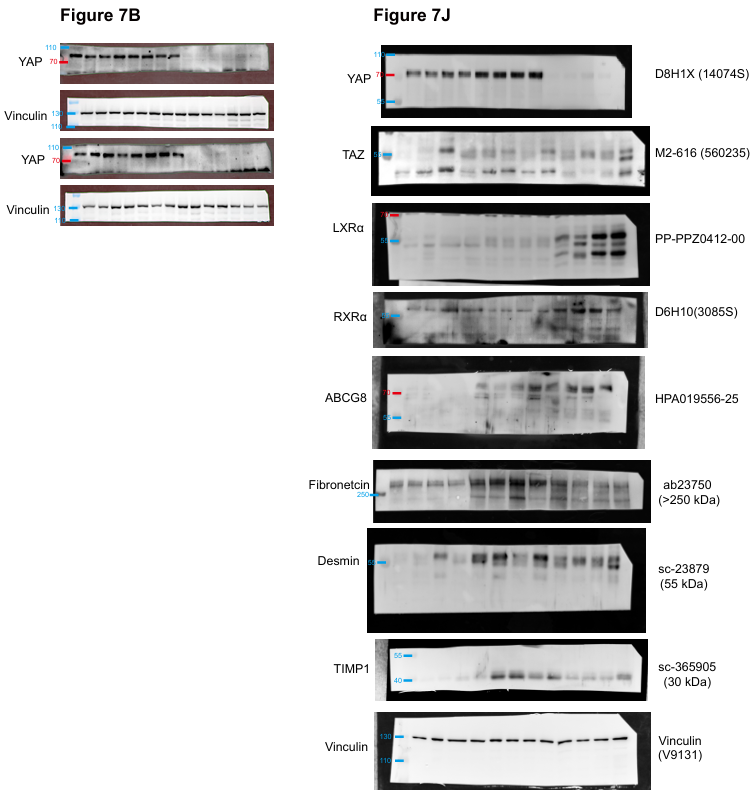
*

**Supplemental Tables**

**Table S1. Baseline characteristics according to the liver stiffness (N = 229)**

|  | **Entire**  (N=229) | **Soft, <5 kPa**  (n=91) | **Medium, 5-8kPa**  (n=87) | **Stiff, >8 kPa**  (n=51) | *P*-value* |
| --- | --- | --- | --- | --- | --- |
| Age, years | 51.8 ± 15.4 | 52.4 ± 13.7 | 42.2 ± 16.0 | 55.3 ± 16.8 | 0.079 |
| Male, n (%) | 118 (51.5) | 48 (52.7) | 49 (56.3) | 21 (41.2) | 0.272 |
| BMI, kg/m^2^ | 27.4 (24.5, 30.2) | 25.9 (23.6, 28.4) | 28.2 (25.0, 30.6) | 28.4 (25.8, 31.9) | 0.363 |
| Diabetes, n (%) | 81 (35.5) | 19 (20.9) | 29 (33.3) | 33 (64.7) | <0.001 |
| Obesity, n (%) | 164 (71.6) | 57 (62.6) | 66 (75.9) | 41 (80.4) | 0.016 |
| AST, IU/L | 34.5 (23.0, 55.8) | 24.0 (21.0, 36.0) | 40.0 (25.0, 65.0) | 53.0 (36.0, 84.0) | 0.008 |
| ALT, IU/L | 38.5 (24.0, 76.0) | 29.0 (19.0, 49.0) | 49.0 (27.8, 91.5) | 63.0 (30.0, 106.0) | 0.265 |
| Total bilirubin, mg/dL | 0.7 (0.6, 1.0) | 0.8 (0.6, 1.0) | 0.7 (0.6, 0.9) | 0.7 (0.6, 1.0) | 0.769 |
| Albumin, g/dL | 4.1 (3.9, 4.3) | 4.1 (3.9, 4.3) | 4.2, (4.0, 4.4) | 4.1 (3.9, 4.3) | 0.019 |
| Platelet, ×10^9^/L | 238 (199, 275) | 242 (211, 276) | 243 (201, 275) | 216 (167, 250) | 0.010 |
| HbA1c, % | 5.9 (5.5, 6.6) | 5.7 (5.4, 6.2) | 6.0 (5.6, 6.6) | 6.5 (5.7, 7.6) | 0.011 |
| Total cholesterol, mg/dL | 181.0 (154.3, 212.0) | 177.0 (150.0, 206.0) | 196.0 (159.5, 216.0) | 172.0 (152.0, 201.0) | 0.067 |
| LDL cholesterol, mg/dL | 105.6 (80.0, 130.5) | 102.0 (77.8, 127.8) | 118.4 (85.5, 134.1) | 102.6 (75.8, 122.6) | 0.072 |
| HDL cholesterol, mg/dL | 44.0 (37.0, 52.8) | 43.0 (37.0, 53.0) | 45.5 (38.0, 52.5) | 42.0 (34.0, 52.0) | 0.259 |
| Triglyceride, mg/dL | 136.0 (95.0, 185.8) | 141.0 (98.0, 176.0) | 131.5 (94.8, 193.0) | 144.0 (93.0, 191.0) | 0.929 |
| Steatosis, n (%) |  |  |  |  |  |
| <5% | 38 (16.6) | 25 (27.5) | 10 (11.5) | 3 (5.9) | <0.001 |
| 5-33% | 58 (25.3) | 25 (27.5) | 22 (25.3) | 11 (21.6) |  |
| 33-66% | 66 (28.8) | 22 (24.2) | 33 (37.9) | 11 (21.6) |  |
| >66% | 67 (29.3) | 19 (20.9) | 22 (25.3) | 26 (51.0) |  |
| Lobular inflammation |  |  |  |  |  |
| Grade 0 | 57 (24.9) | 37 (40.7) | 16 (18.4) | 4 (7.8) | <0.001 |
| Grade 1 | 124 (54.1) | 45 (49.5) | 48 (55.2) | 31 (60.8) |  |
| Grade 2 | 45 (19.7) | 9 (9.9) | 21 (24.1) | 15 (29.4) |  |
| Grade 3 | 3 (1.3) | 0 | 2 (2.3) | 1 (2.0) |  |
| Ballooning |  |  |  |  |  |
| Grade 0 | 113 (49.3) | 69 (75.8) | 33 (37.9) | 11 (21.6) | <0.001 |
| Grade 1 | 103 (45.0) | 22 (24.2) | 51 (58.6) | 30 (58.8) |  |
| Grade 2 | 13 (5.7) | 0 | 3 (3.4) | 10 (19.6) |  |
| Fibrosis |  |  |  |  |  |
| Stage 0 | 53 (23.1) | 37 (40.7) | 13 (14.9) | 3 (5.9) | <0.001 |
| Stage 1 | 112 (48.9) | 45 (49.5) | 54 (62.1) | 13 (25.5) |  |
| Stage 2 | 45 (19.7) | 9 (9.9) | 18 (20.7) | 18 (35.3) |  |
| Stage 3 | 7 (3.1) | 0 | 1 (1.1) | 6 (11.8) |  |
| Stage 4 | 12 (5.2) | 0 | 1 (1.1) | 11 (21.6) |  |
| LSM, kPa | 5.5 (4.4, 7.6) | 4.2 (3.5, 4.5) | 6.0 (5.4, 6.8) | 11.7 (9.9, 16.0) | <0.001 |
| CAP | 294.0 (254.3, 329.0) | 276.0 (237.0, 313.0) | 306.0 (262.0, 340.0) | 304.5 (274.0, 339.0) | 0.986 |

The data are expressed as means ± standard deviations for normally distributed variables or medians (interquartile range) if not normally distributed.

^*^From the independent ANOVA test, Kruskal-Wallis test or Chi-square test to compare 3 subgroups.

**Table S2. Baseline characteristics according to the liver stiffness (N = 34)**

|  | **Entire**  (N= 34) | **Soft, <5 kPa**  (n= 12) | **Medium, 5-8 kPa**  (n=9) | **Stiff, >8 kPa**  (n=13) | *P*-value* |
| --- | --- | --- | --- | --- | --- |
| Age, years | 51.4 ± 16.6 | 51.9 ± 13.0 | 44.6 ± 17.8 | 55.7 ± 18.2 | 0.307 |
| Male, n (%) | 15 (44.1) | 6 (50.0) | 4 (44.4) | 5 (38.5) | 0.567 |
| BMI, kg/m^2^ | 28.3 (24.7, 32.9) | 25.2 (23.5, 31.7) | 31.2 (26.9, 33.4) | 28.9 (25.2, 34.2) | 0.713 |
| Diabetes, n (%) | 11 (32.4) | 1 (8.3) | 2 (22.2) | 8 (61.5) | 0.005 |
| Obesity, n (%) | 24 (70.6) | 6 (50.0) | 8 (88.9) | 10 (76.9) | 0.154 |
| AST, IU/L | 30.0 (22.0, 72.0) | 21.5 (18.3, 24.8) | 30.0 (22.5, 50.0) | 72.0 (45.5, 85.0) | 0.025 |
| ALT, IU/L | 37.0 (22.0, 74.0) | 27.5 (15.3, 46.8) | 33.0 (20.0, 134.5) | 63.0 (36.0, 121.0) | 0.422 |
| Total bilirubin, mg/dL | 0.7 (0.6, 0.9) | 0.9 (0.6, 1.0) | 0.6 (0.5, 0.7) | 0.7 (0.6, 1.0) | 0.023 |
| Albumin, g/dL | 4.1 (3.9, 4.3) | 4.2 (4.0, 4.3) | 4.1 (3.8, 4.4) | 4.0 (3.9, 4.2) | 0.635 |
| Platelet, ×10^9^/L | 243 (211, 278) | 256 (217, 272) | 278 (260, 291) | 199 (164, 234) | 0.010 |
| HbA1c, % | 6.0 (5.5, 7.2) | 5.8 (5.4, 6.0) | 5.7 (5.6, 6.3) | 7.6 (5.7, 8.4) | 0.052 |
| Total cholesterol, mg/dL | 200.0 (162.0, 218.0) | 175.5 (159.9, 228.5) | 214.0 (200.0, 216.0) | 184.0 (155.0, 226.5) | 0.367 |
| LDL cholesterol, mg/dL | 118.0 (82.0, 143.4) | 105.0 (78.9, 139.5) | 130.6 (124.3, 146.4) | 114.2 (63.5, 146.2) | 0.171 |
| HDL cholesterol, mg/dL | 46.5 (40.0, 57.0) | 48.5 (37.5, 53.0) | 46.0 (41.0, 53.5) | 45.0 (41.0, 65.0) | 0.841 |
| Triglyceride, mg/dL | 136.0 (108.0, 180.0) | 156.0 (120.8, 185.0) | 136.0 (119.5, 161.5) | 109.0 (69.5, 207.5) | 0.404 |
| Steatosis, n (%) |  |  |  |  |  |
| <5% | 3 (8.8) | 2 (16.7) | 0 | 1 (7.7) | 0.083 |
| 5-33% | 7 (20.6) | 3 (25.0) | 2 (22.2) | 2 (15.4) |  |
| 33-66% | 14 (41.2) | 5 (41.7) | 6 (66.7) | 3 (23.1) |  |
| >66% | 10 (29.4) | 2 (16.7) | 1 (11.1) | 7 (53.8) |  |
| Lobular inflammation |  |  |  |  |  |
| Grade 0 | 7 (20.6) | 4 (33.3) | 2 (22.2) | 1 (7.7) | 0.028 |
| Grade 1 | 18 (52.9) | 7 (58.3) | 5 (55.6) | 6 (46.2) |  |
| Grade 2 | 7 (20.6) | 1 (8.3) | 1 (11.1) | 5 (38.5) |  |
| Grade 3 | 2 (5.9) | 0 | 1 (11.2) | 1 (7.7) |  |
| Ballooning |  |  |  |  |  |
| Grade 0 | 13 (38.2) | 8 (66.7) | 3 (33.3) | 2 (15.4) | 0.003 |
| Grade 1 | 18 (52.9) | 4 (33.3) | 6 (66.7) | 8 (61.5) |  |
| Grade 2 | 3 (8.8) | 0 | 0 | 3 (23.1) |  |
| Fibrosis |  |  |  |  |  |
| Stage 0 | 4 (11.8) | 4 (33.3) | 0 | 0 | <0.001 |
| Stage 1 | 14 (41.2) | 6 (50.0) | 6 (66.7) | 2 (15.4) |  |
| Stage 2 | 10 (29.4) | 2 (16.7) | 3 (33.3) | 5 (38.5) |  |
| Stage 3 | 3 (8.8) | 0 | 0 | 3 (23.1) |  |
| Stage 4 | 3 (8.8) | 0 | 0 | 3 (23.1) |  |
| LSM, kPa | 6.2 (4.5, 11.1) | 4.4 (3.7, 4.6) | 6.1 (5.5, 6.7) | 14.8 (10.4, 19.9) | <0.001 |
| CAP | 311.0 (266.0, 342.0) | 289.0 (276.0, 343.0) | 313.0 (262.5, 345.0) | 315.0 (222.5, 344.5) | 0.616 |

The data are expressed as means ± standard deviations for normally distributed variables or medians (interquartile range) if not normally distributed.

^*^From the independent ANOVA test, Kruskal-Wallis test or Chi-square test to compare 3 subgroups

| **Table S3. Oligonucleotides used in the study** | | |
| --- | --- | --- |
| **Gene symbol** | **Forward** | **Reverse** |
| **Human qPCR primers** | | |
| *hGAPDH* | GTCTCCTCTGACTTCAACAGCG | ACCACCCTGTTGCTGTAGCCAA |
| *hCTGF* | CCGTACTCCCAAAATCTCCA | GTAATGGCAGGCACAGGTCT |
| *hCYP8B1* | CTGGAGACCAAGCAGTCCTTTG | GATACTCCTGCCCACTGGACAT |
| *hABCG5* | GATTGTCGTCCTCCTGGTGGAA | TCTCCGAAGCTCAGGATGGCAA |
| *hCYP27A1* | GTGCTGCCTTTCTGGAAGCGAT | TAGCCAGACACCTGGATGCCAT |
| *hACACA* | AGTGGGTCACCCCATTGTT | TTCTAACAGGAGCTGGAGCC |
| *hCYP7A1* | CAAGCAAACACCATTCCAGCGAC | ATAGGATTGCCTTCCAAGCTGAC |
| **Mouse qPCR primers** | | |
| *mActb* | CTGAGAGGGAAATCGTGCGT | TGTTGGCATAGAGGTCTTTACGG |
| *mGapdh* | AGGTCGGTGTGAACGGATTTG | GGGGTCGTTGATGGCAACA |
| *mCyr61* | TAAGGTCTGCGCTAAACAACTC | CAGATCCCTTTCAGAGCGGT |
| *mCtgf* | GGCCTCTTCTGCGATTTCG | GCAGCTTGACCCTTCTCGG |
| *mAbcg5* | TGCCATCCTGACTTACGGAGAG | CTGCTTTGGGTGTCCACTGATG |
| *mAbcg1* | GACACCGATGTGAACCCGTTTC | GCATGATGCTGAGGAAGGTCCT |
| *mAbcg8* | GGTCCTTCTGATGACATCTGGC | CGTCTGTCGATGCTGGTCAAGT |
| *mAbca1* | AGTGATAATCAAAGTCAAAGGCACAC | AGCAACTTGGCACTAGTAACTCTG |
| *mCyp7a1* | AGCAACTAAACAACCTGCCAGTACTA | GTCCGGATATTCAAGGATGCA |
| *mCyp8b1* | GGCTGGCTTCCTGAGCTTATT | ACTTCCTGAACAGCTCATCGG |
| *mSrebf2* | AGAAAGAGCGGTGGAGTCCTTG | GAACTGCTGGAGAATGGTGAGG |
| *mLdlr* | GAATCTACTGGTCCGACCTGTC | CTGTCCAGTAGATGTTGCGGTG |
| *mHmgcr* | GCTCGTCTACAGAAACTCCACG | GCTTCAGCAGTGCTTTCTCCGT |
| *mHmgcs* | GGAAATGCCAGACCTACAGGTG | TACTCGGAGAGCATGTCAGGCT |
| *mNr1h3* | TGGGATGTCCACGAGTGACTGTTT | TCCCTTAATGCTACGGAAGGCTCT |
| *mAcaca* | GTTCTGTTGGACAACGCCTTCAC | GGAGTCACAGAAGCAGCCCATT |
| *mSrebf1* | AACGTCACTTCCAGCTAGAC | CCACTAAGGTGCCTACAGAGC |
| *mScd1* | TTCTTGCGATACACTCTGGTGC | CGGGATTGAATGTTCTTGTCGT |
| *mFasn* | GCTGCGGAACTTCAGGAAAT | AGAGACGTGTCACTCCTGGACTT |
| *mApoE* | ACAGATCAGCTCGAGTGGCAAA | ATCTTGCGCAGGTGTGTGGAGA |
| *mTimp1* | TCTTGGTTCCCTGGCGTACTCT | GTGAGTGTCACTCTCCAGTTTGC |
| *mCol1a1* | GGTCTTGGTGGTTTTGTATTCG | AACAGTCGCTTCACCTACAGC |
| **siRNAs** | | |
| *mNr1h3*_1 | GACCUUGUUGACCCAACCAAAUAaa | UUUAUUUGGUUGGGUCAACAAGGUCUU |
| *mNr1h3*_2 | GAAGACCUCUGCAAUCGAGGUCAtg | CAUGACCUCGAUUGCAGAGGUCUUCAG |
| **ChIP primers** | | |
| *ABCA1* | CCCAGCTTCCCCATCTGCGC | CCGGAGGTGGGGTGCCCAAT |
| *ABCG1* | TTCTGTGGACAGGTACTAGGT | CCACAAACATAGGTAGTCCAG |
| *ABCG5* | TGGACCAGGCAGATCCTCAAA | CCGTTCACATACACCTCCCC |
| **Guide RNAs** | |  |
| *hLATS1* | CGTGCAGCTCTCCGCTCTAA |  |
| *hLATS2* | TACGCTGGCACCGTAGCCCT |  |
| *hYAP* | CATCAGATCGTGCACGTCCG |  |
| *hTAZ* | TGTCTAGGTCCTGCGTGACG |  |

| **Table S4. Antibodies used in the study.** | | | |  |
| --- | --- | --- | --- | --- |
| Target | Supplier | Application | Product reference | RRID |
| YAP | Cell Signaling Technology | WB | 14074 | AB_2650491 |
| YAP/TAZ |  | IF | 8418 | AB_10950494 |
| HA-Tag |  | WB, IP | 2367 | AB_10691311 |
| RXRα |  | WB, IP, ChIP | 3085 | AB_11140620 |
| Normal Rabbit IgG |  | ChIP | 2729 | AB_1031062 |
| SREBF2 | BD Biosciences | WB | 557037 | AB_396560 |
| TAZ |  | WB | 560235 | AB_1645338 |
| CYP7A1 | Merck | WB | MABD42 | AB_2756360 |
| HMGCR | Invitrogen | WB | PA5-37367 | AB_2554032 |
| Vinculin | Sigma | WB | V9131 | AB_477629 |
| LXRα | R&D Systems | WB, IP, ChIP | PP-PPZ0412-00 | AB_2154888 |
| Active YAP1 | Abcam | IHC | ab205270 | AB_2813833 |
| Fibronectin |  | WB | ab23750 | AB_447655 |
| Human ABCG8 | Atlas Antibodies | WB, IHC | HPA019556 | AB_1844444 |
| c-Myc | Santa Cruz Biotechnology | WB, IP | sc-40 | AB_627268 |
| YAP |  | IF | sc-101199 | AB_1131430 |
| Desmin |  | WB | sc-23879 | AB_627416 |
| TIMP1 |  | WB | sc-365905 | AB_10848565 |
